# Formation of the Codon Degeneracy during Interdependent Development between Metabolism and Replication

**DOI:** 10.3390/genes12122023

**Published:** 2021-12-20

**Authors:** Dirson Jian Li

**Affiliations:** Ministry of Education Key Laboratory for Non-Equilibrium Synthesis and Modulation of Condensed Matter, Shaanxi Province Key Laboratory of Advanced Functional Materials and Mesoscopic Physics, School of Physics, Xi’an Jiaotong University, Xi’an 710049, China; dirson@xjtu.edu.cn

**Keywords:** the codon degeneracy, coevolution of tRNAs with aaRSs, relative stability of triplex base pair, the evolution of the genetic code

## Abstract

Nirenberg’s genetic code chart shows a profound correspondence between codons and amino acids. The aim of this article is to try to explain the primordial formation of the codon degeneracy. It remains a puzzle how informative molecules arose from the supposed prebiotic random sequences. If introducing an initial driving force based on the relative stabilities of triplex base pairs, the prebiotic sequence evolution became innately nonrandom. Thus, the primordial assignment of the 64 codons to the 20 amino acids has been explained in detail according to base substitutions during the coevolution of tRNAs with aaRSs; meanwhile, the classification of aaRSs has also been explained.

## 1. Introduction

The difficulty in the field of the origin of the genetic code is due to the lack of key experiments to reproduce the primordial scenario of evolution of life. The debate about the nature of life even makes it difficult to reach a consensus on the definition of life. Pragmatically, we need to put together the few following well-established and enlightening observations to have a deep insight into the transition from non-living to living phenomena. Wong values that Phase I amino acids appeared earlier than Phase II amino acids in prebiotic evolution [1,2]. Pouplana and Schimmel prefer aminoacyl-tRNA synthetases (aaRSs) to clues to establishment of the genetic code [3,4]. Woese divided cellular life into three domains [5], which helps to comprehend last universal common ancestor (LUCA). In addition, a living JCVI-syn1.0 cell has been created by combining a cytoplasm without natural DNA and a chemically synthesised chromosome with Venter’s watermark [6]. Verily, potential contradictions, as will be explained next, have yet appeared in the few above common sense observations, which urges us to be serious in collecting experimental observations and extremely cautious in interpreting them.

Pouplana and Schimmel have overlooked the above two phases of amino acids. By comparing sequences and structures, the 20 aaRSs are divided into two distinct classes, each of which is subdivided into three subclasses. Pouplana and Schimmel assumed simultaneous association of two aaRSs on a single tRNA to interpret the symmetrical subclasses between the two classes of aaRSs, where the aaRS pairs, namely IleRS (subclass Ia) and ThrRS (IIa), GlnRS (Ib) and AspRS (IIb), and TyrRS (Ic) and PheRS (IIc), can cover the tRNA acceptor stem without major steric clashes and, meanwhile, link together the specific subclasses. However, Gln as a Phase II amino acid recruited much later than Asp as a Phase I amino acid [7,8]. It becomes suspicious to associate GlnRS and AspRS on a single tRNA simultaneously.

The creation of JCVI-syn1.0 is quite different from the primordial picture for supposed LUCA, where the former was synthesised rapidly, while the latter evolved during a long period. Moreover, a new cell can be certainly recreated anytime in JCV institute if a synthesised cell dies, but the evolution of life has to be halted if LUCA died. Life is a phenomenon rather than eternal matter, which emerged from interdependent development between metabolism and replication. The cell JCVI-syn1.0 was created by combining a cytoplasm without natural DNA and a chemically synthesised chromosome, whose life was acquired by integrating metabolism in cytoplasm and replication of chromosome, so JCVI-syn1.0 belongs to “cell without living parents” (CwoP). The apparatuses in JCV institute can play the role of “‘the Hand that feeds you’ for CwoPs” (HfC). In such an “HfC-CwoP” mechanism, the long-lasting non-living HfC is able to rapidly create various ephemeral living CwoPs at any time. The successful creation of the cell JCVI-syn1.0 is just a contemporary transition from non-living to living phenomena, whose mechanism can enlighten the prebiotic transition from non-living to living phenomena. LUCA followed Darwin’s vague idea of common ancestor. Many popular theories have yet forgotten to explain the viability of simple LUCA during the geologically long period. In fact, only living LUCA can hardly survive such a geologically long period. However, an “HfC-CwoP”-like mechanism is feasible to bridge the gap between the nonliving and living by continually generating viable systems, where an auxiliary non-living HfC evolved during the geologically long period to create various living CwoPs at any appropriate time. It is logical to assume that the molecular ancestors, at different times, had the same chemical characteristics given the same source of primordial matter. Thus, death of an individual CwoP and extinction in its offsprings cannot interrupt the process of evolution of life. When numerous CwoPs and their offsprings gathered and formed into a continuously evolving ecosystem, the HfC-like apparatus stepped down and vanished.

Nirenberg’s genetic code chart [9] revealed a profound correspondence between amino acids and codons. It is still a mystery how the codon degeneracy formed yet. If introducing an HfC-like apparatus during the evolution of tRNAs with aaRSs, the symmetrical subclasses of aaRSs can also be explained without the above redundant complex of two aaRSs on a single tRNA. The crux in the coevolution of aaRSs and tRNAs is how to continually generate and maintain non-random sequences in the geologically long period. In this paper, I propose that certain primordial polymer molecules played the role of HfC in the prebiotic evolution. Triple-helical nucleic acids provide a possible picture. Hence, non-random sequences were generated routinely based on the triplex base substitutions, where low stable triplex bases were substituted spontaneously by more stable triplex base pairs [10,11]. Numerous non-random DNA sequences were generated, along which aaRSs and tRNAs coevolved. Remarkably, the evolution of complementary strands also accounts for the symmetrical subclasses of aaRSs. This process is intricate but elegant; thus, both the codon degeneracy and the two classes, as well as their symmetrical subclasses of aaRSs, have been explained thoroughly.

The interesting property of triplex base pairs in triple-helical nucleic acids has been often ignored in the field of the origin of life. In experiments, the homopurine and homopyrimidine strands tend to form triple helix [10,12,13] besides double helix. It is not unreasonable to introduce triplex nucleic acids in the prebiotic evolution, considering the substantial role of triplex DNAs within recA fibers in the fundamental process of recombination. Furthermore, the triplex base pairs remained in tRNAs [14] also indicate that the origin of tRNAs depended on stability of triplex base pairs, which hints the rationality of this scenario. The prebiotic reciprocal impact between the HfC-like triplex DNAs and the CwoP-like systems with evolving genetic code is essential for the emergence of life where the evolution of prebiotic informative molecules was driven by the triplex DNAs whose evolution in return needed the help of prebiotic informative molecules, which is analogous to the reciprocal impact between Wilkinson’s boring machine and Watt’s steam engine in the industrial revolution, where a boring machine was driven by the steam engine whose improved cylinder in return needed to be bored by a boring machine. In such an interim scenario, the “chicken-egg” problem becomes an opportunity rather than a dilemma that RNA world theory tends to avoid.

The synthesis of adenine from hydrogen cyanide by Oro initiated the prebiotic chemistry of nucleic acids [2,15]. HCN tetramer is polymerised to a intractable solid, from which adenine and guanine can be recovered [16]. There are also HCN-independent routes of purine synthesis [17]. Cyanoacetylene, via electrically discharging nitrogen and methane, reacts with cyanic acid to give cytosine. Hydrolysis of cytosine yields uracil [16,18,19]. Progress in the prebiotic synthesis of the pyrimidine ribonucleosides [20], together with recent advances in non-enzymatic RNA replication [21], have given credence to the RNA world theory. So far, progress towards the abiotic synthesis of purine nucleosides has to use disputable starting materials [22]. The nature of the first genetic polymer is the subject of major debate. A prebiotic scenario for coexistence and co-evolution of RNA and DNA has been investigated [23,24]. Synthesis under prebiotic conditions gives credence to the idea that DNA could appear concurrently with RNA, instead of being its later descendent [25]. Purine deoxyribonucleosides and pyrimidine ribonucleosides may have coexisted before the emergence of life [26]. Resently, Xu et al. demonstrated a high-yielding, completely stereo-, regio-, and furanosyl-selective prebiotic synthesis of the purine deoxyribonucleosides, leading to a mixture of deoxyadenosine, deoxyinosine, cytidine, and uridine [27]. Considering that the homopurine and homopyrimidine RNA and DNA strands tend to form triple helix [10], substitutions of triplex base pairs among the prebiotic triplex nucleic acids also contribute to the prebiotic evolution.

Although numerous theories have attempted to explain the origin of the genetic code in literature [3,28], a candidate theoretical framework must at least be able to explain: (i) the driving force in the prebiotic sequence evolution, (ii) the degeneracies 6, 4, 3, 2, and 1 for the respective 20 amino acids, and (iii) the two classes of aaRSs to recognise tRNAs from either major or minor groove sides. These are the tasks of this article. I found that the evolution of triple-helical nucleic acids driven by the spontaneous substitutions of triplex base pairs provides an elegant roadmap picture for the prebiotic evolution. Accordingly, the assignment of the 64 codons to the 20 amino acids has been explained one by one based on the coevolution of aaRSs and tRNAs, where the symmetrical subclasses of aaRSs need the help of palindromic para-codons. There are many profound and amazing relationships among traditionally separate fields, summarised as follows. The coevolution of tRNAs with aaRSs along the roadmap that is established by the relative stabilities of triplex base pairs [10,11] agrees with both the codon degeneracy [29,30] and two classes of aaRSs [31] in observations. The earliest amino acids recruited in the initiation stage of the roadmap agrees with phase I amino acids in Miller-Urey experiment [32] and carbonaceous chondrites [33,34,35], and see Chapter 6 in [2]. The recruitment orders of amino acids and codons on the roadmap agrees with the variation trends of amino acid frequencies in proteomes [36] and codon position GC content variation [37]. The expansion of codons along the roadmap agrees with biosynthetic families of amino acids [1]. All the above agreements between predictions and observations prompted the formation of the present hypothesis on the origin of the genetic code.

## 2. Materials and Methods

### 2.1. Triplex Picture

**Figure 1:**
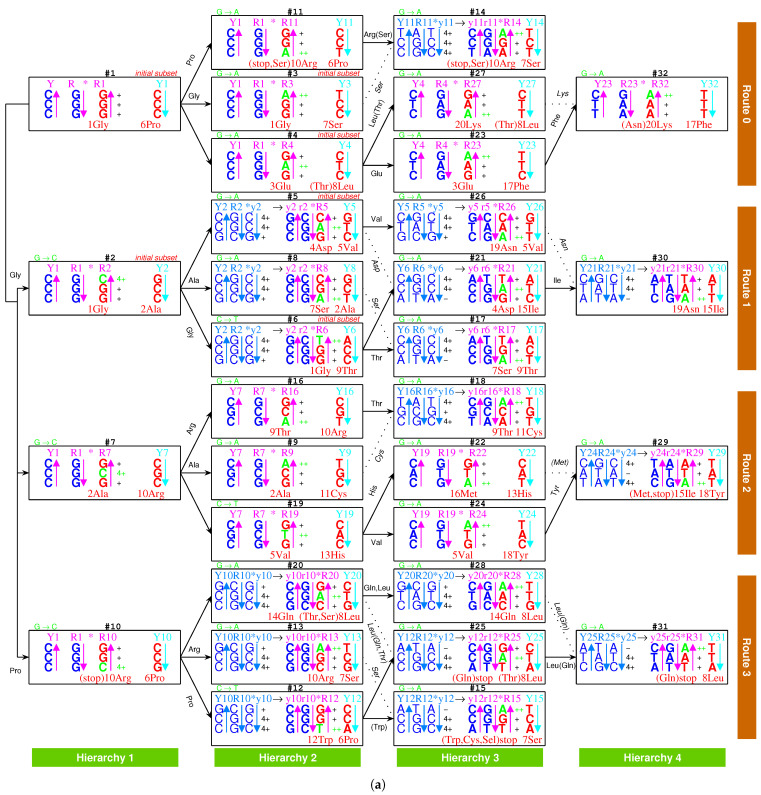

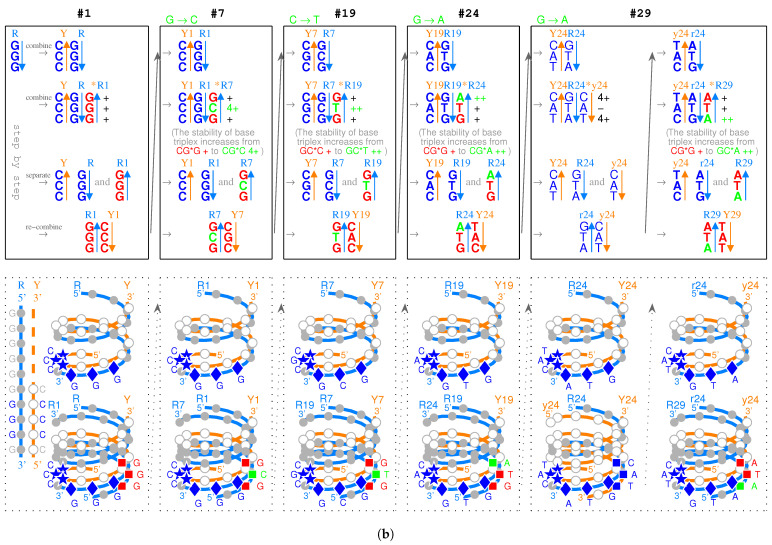
The origin of the genetic code. (**a**) The roadmap for the evolution of the genetic code. The 64 codons formed from base substitutions in triplex DNAs are in red. Only three-base-length segments of the triplex DNAs are shown explicitly; the whole length right-handed triplex DNAs are indicated in Figure 1b. In each position #n (n=1,2,⋯,32), the #n codon pair on Rn, and Yn is in red. The relative stabilities of the triplex base pairs (−, +, ++, 4+) are written to the right of the base triplexes, where the increased relative stabilities of triplex base pairs in base substitutions are indicated in green. Each triplex DNA is denoted by three arrows, whose directions are from 5’ to 3’. The YR∗R triplex DNAs are in pink, and the YR∗Y triplex DNAs in azure. The recruitment order of codon pairs are from #1 to #32, and the recruitment order of the 20 amino acids are to the left of them, respectively. Non-standard genetic codes are indicated by brackets beside the corresponding amino acids. The Route0−3 and Hierarchy1∼4 are indicated to the right of and below the roadmap, respectively. The evolution of the genetic code are denoted by black arrows, beside which pair connections are indicated by the corresponding amino acids. Refer to an example in Figure 1b to understand details of the roadmap; refer to Figure 2 to understand the critical role of relative stabilities of triplex base pairs in achieving the real genetic code; refer to Figure 5a,b to see the origin of tRNAs; refer to Figure 3a to see the coherent relationship between the recruitment orders of codons and amino acids; refer to Figure 3b to see the codon degeneracy in the symmetric roadmap. (**b**) A detailed description of the roadmap (see Supplementary Movie S1). Taking, for example, from #1 to #29, the evolution of the genetic code from #1, to #7, to #19, to #24, and, at last, to #29 are explained in detail in the upper boxes, and the corresponding right-handed single-stranded, double-stranded, and triple-stranded DNAs are shown in the lower boxes, respectively.

The genetic code is a common and essential feature of life, which can be regarded as a relic of the prebiotic emergence of informative molecules. The complexity of the problem for the origin of the genetic code may exceed all the theoretical estimations, such as frozen accident, error minimisation, stereochemical interaction, amino acid biosynthesis, expanding codons, etc. [1,38,39,40,41,42,43,44,45,46,47,48]. So far, it can hardly describe the evolution of the genetic code step by step so as to explain the formation of the codon degeneracy in detail. Here, a triplex picture is proposed to describe the intricate evolution of the genetic code thoroughly, by which both the formation of the codon degeneracy and the classification of aaRSs have been explained in a same theoretical framework. The complexity of the following explanation of the codon degeneracy is comparable to that of a symphony score. The simplest method of score-reading is to concentrate on an individual voice part that can be heard particularly well and then going over to section-by-section or selective reading. Similarly, here are some suggestions for reading the following technical explanation of the codon degeneracy in the triplex picture. Please watch the Supplementary Movie S1 and start from Figure 1 and then figures on tRNAs and aaRSs so as to understand the recruitment of the 20 amino acids during coevolution of tRNAs with aaRSs.

Guessing the right prebiotic picture is the key for understanding the origin of the genetic code. Here, I propose a triplex picture for the prebiotic sequence evolution. There are 8 kinds of triplex nucleic acids $S\cdot S^{\prime }*S^{\prime \prime }$ (‘·’ represents a Watson-Crick base pair, while ‘∗’ a Hoogsteen base pair), where the strands $S,S^{\prime },S^{\prime \prime }$ can be either DNA or RNA [49,50,51], such as the triplex DNA D·D∗D and the triplex nucleic acids mixed with DNA and RNA D·D∗R, etc. The YR∗R triplex DNA PolyC·PolyG∗PolyG is supposed as the initial physical conditions for the evolution of the genetic code. The 64 codons have been recruited one by one with the D·D∗D sequence evolution by alternative separation and recombination of the three strands in the periodic changing environments. Such sequence evolution in the prebiotic evolution was driven by the substitutions of triplex base pairs according to their relative stabilities. The sequence evolution of D·D∗D led to the evolution of the genetic code, while the RNA strands separated from the coevolving D·D∗R yielded tRNAs and the template RNAs for aaRSs. The tRNAs and aaRSs were generated in accompany with the recruitment of the corresponding codons, respectively. So, the triplex picture gives a physical basis for the coevolution of the genetic code with the corresponding tRNAs and aaRSs.

#### Nomenclature and Notation

- Notations for the 20 amino acids, 20 aaRSs, and the corresponding tRNAs (n=1to20): amino acid No.n↔aaRSn↔ tRNA tn, $tn^{\prime }$, $tn^{+}$, $tn^{+}{}^{\prime }$, $tn^{-}$, $tn^{-}{}^{\prime }$, where the amino acids from No.1 to No.20 are, respectively, as follows: 1Gly, 2Ala, 3Glu, 4Asp, 5Val, 6Pro, 7Ser, 8Leu, 9Thr, 10Arg, 11Cys, 12Trp, 13His, 14Gln, 15Ile, 16Met, 17Phe, 18Tyr, 19Asn, 20Lys, and aaRSn are, respectively, as follows: aaRS1 (namely GlyRS), aaRS2 (namely AlaRS), and so on.
- Triplex DNAs (D·D∗D): YR∗R, YR∗Y and the inverse triplex DNAs: yr∗r, yr∗y, where *Y*, *y* stands for pyrimidine strands, and *R*, *r* purine strands.
- Triplex DNA·DNA*RNA (D·D∗R): $yr*r_{t}$, $yr*y_{t}$, $YR*R_{t}$, $YR*Y_{t}$, where two types of tRNAs can be generated by linking the RNA strands $5^{\prime }y_{t}+r_{t}3^{\prime }$ or $5^{\prime }R_{t}+Y_{t}3^{\prime }$, and aaRSs can approach tRNAs from major groove side (M) or minor groove side (m).
- Codon pairs: #1GGG·CCC, etc.; pair connections: #1−Gly−#2, etc.; route dualities: #1−Gly−#3∼#2−Gly−#6, etc., where the numbers #m (m=1to32) indicates the positions on the roadmap

### 2.2. Origin of the Genetic Code

#### 2.2.1. The Roadmap

In the triplex picture, I obtained a roadmap for the evolution of the genetic code (or the roadmap for short). The validity of the roadmap depends essentially on the experimental data of triplex base pairs. The stabilities of the 16 triplex base pairs in triplex DNA are listed from instability (−), weak (+) to strong (++, 3+, 4+) as follows [10,11]:

$$\begin{array}{ccc} \left(-\right) & & GC*A,\;AT*C,\;AT*A\\ \left(+\right) & & CG*G,\;TA*C,\;TA*A,\;TA*G,\;GC*C,\;GC*G,\;AT*T\\ \left(++\right) & & CG*A,\;CG*T,\;GC*T\\ \left(3+\right) & & AT*G\\ \left(4+\right) & & CG*C,\;TA*T. \end{array}$$

The above stability order in experiments played a significant role in the primordial evolution of triplex DNA. The substitutions of triplex base pairs from weak to strong provided the principal driving force in the prebiotic sequence evolution.

**Figure 2:**
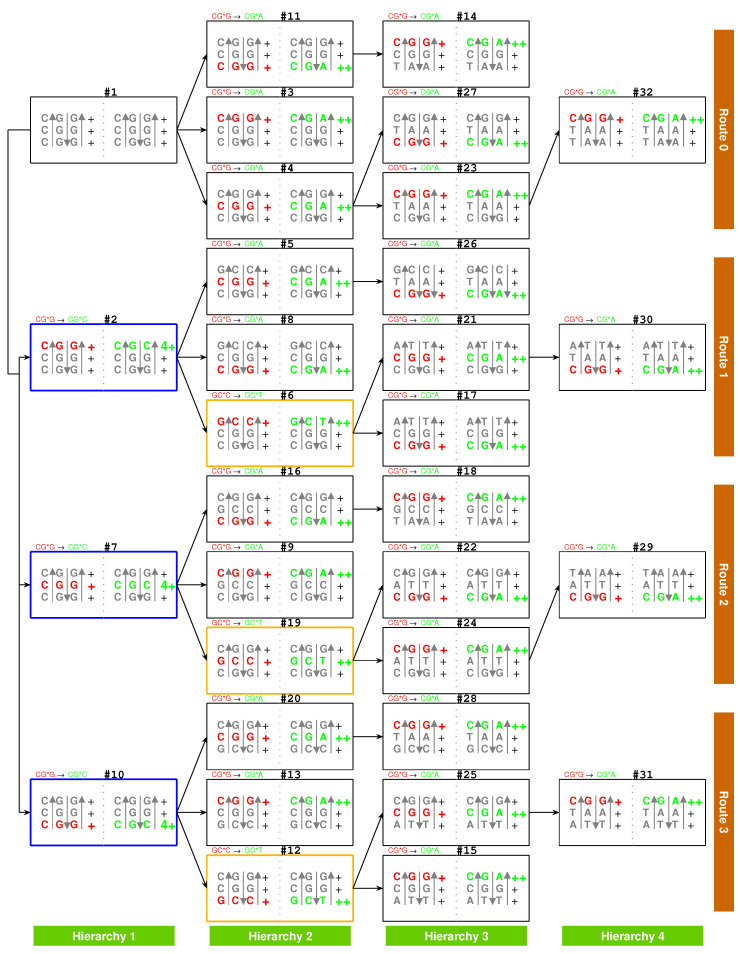
The driving force in the evolution of the genetic code based on the relative stabilities of triplex base pairs. The base substitutions on the roadmap occur when the relative stabilities of triplex base pairs increase. The roadmap is the best result to avoid the unstable triplex base pairs. So, the universal genetic code is a narrow choice by the relative stabilities of triplex base pairs. The relative stability increases from (+) of the triplex base pair CG∗G to (4+) of the triplex base pair CG∗C at #2, #7, and #10 that initiates Route1∼3, respectively. GC∗C (+) changes to GC∗T (++) at #6, #19, and #12, and CG∗G (+) changes to CG∗A (++) at other positions on the roadmap.

**Figure 3:**
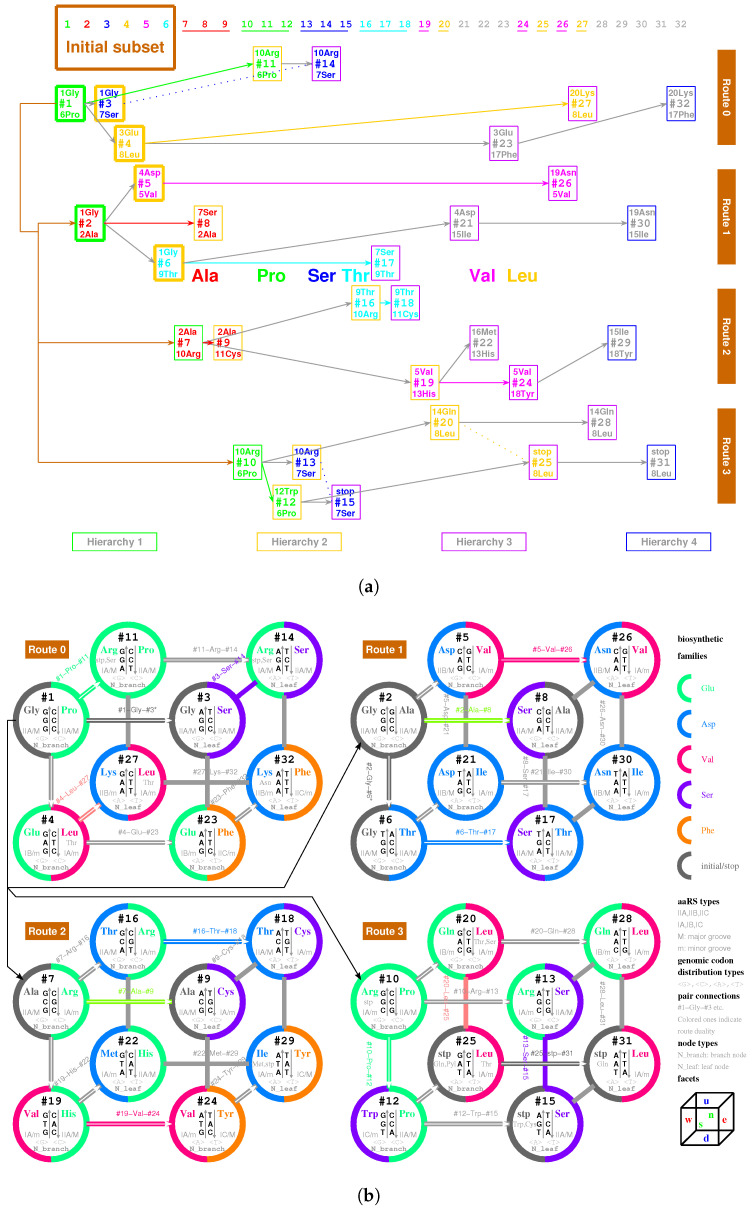
(**a**) Cooperative recruitment of codons and amino acids. Codon pairs are plotted from left to right according to their recruitment order. The initial subset plays a crucial role in the expansion of the genetic code along the roadmap. The 6 biosynthetic families of the amino acid are distinguished by different colours. (**b**) The cubic roadmap. This is a revised roadmap Figure 1a to indicate the symmetry in the evolution of the genetic code. The four routes are represented by four cubes, respectively. Pair connections are marked besides the evolutionary arrows. Route dualities are indicated by same colours for the corresponding pair connections.

**Figure 4:**
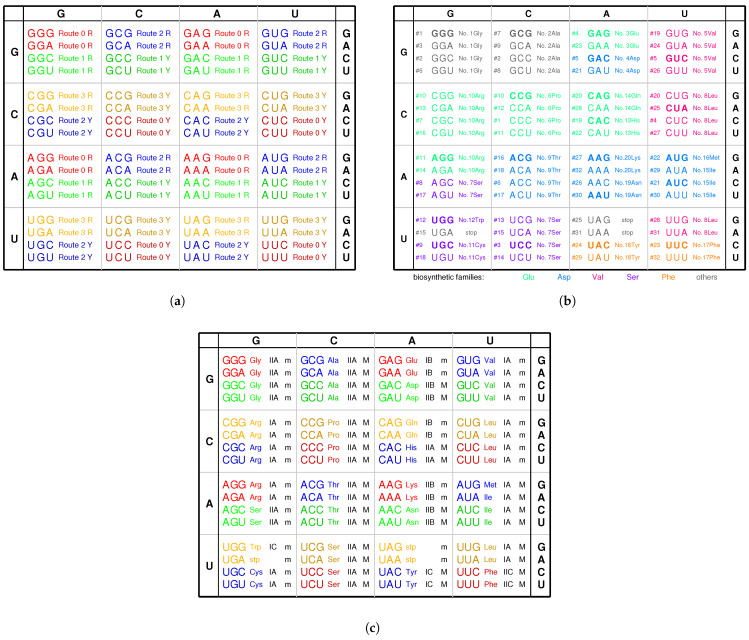
(**a**) The distribution of codons from R- and Y-strands of Route0−3 in the GCAU genetic code table. The pattern of the 4×4 codon boxes for the degenerate codons relates to such a distribution of the four routes, owing to the evolution of the genetic code along the roadmap. (**b**) The GCAU genetic code table. The clusterings of biosynthetic families (Glu, Asp, Val, Ser, Phe) in the GCAU genetic code table. Such nice clusterings are correspondingly observed in the R- and Y-strands of Route0−3 in Figure 3b (denoted in the same group of colour as in the present figure). The clusterings of biosynthetic families in the present figure are closely related to the distribution of codons from R- and Y-strands of Route0−3, owing to the recruitment of amino acids along the roadmap. Generally speaking, the amino acids are arranged properly in the recruitment order from No.1 to No.20 along the direction from *G*, *C* to *A*, *U* in the GCAU genetic code table. (**c**) The distribution of types of aaRSs in the GCAU genetic code table. The aaRSs can be divided into ClassII and ClassI, which can be divided into subclasses IIA, IIB, IIC and IA, IB, IC, respectively. The aaRSs can also be divided into minor groove ones (*m*) and major groove ones (*M*).

At the beginning of the evolution of the genetic code, there existed single-stranded DNA PolyG and PolyC, which tended to form a triplex DNA (Figure 1a,b) [10,13]. PolyC·PolyG∗PolyG is a usual YR∗R triplex DNA, which is combined by triplex base pair CG∗G (Figure 1b and Supplementary Movie S1). The sequences evolved via substitutions of triplex base pairs in the procedure of alternative combining and separating for the strands of triple-stranded DNA. Only three kinds of substitutions of triplex base pairs are practically required on the roadmap: (1) substitution of (+)CG∗G by (++)CG∗A [10,11], with the transition from *G* to *A* in the third *R* strand. This is of the most common substitution on the roadmap by which all the codons in Route0 and most codons in Route1∼3 were recruited (Figure 1a); (2) substitution of (+)CG∗G by (4+)CG∗C, with the transversion from *G* to *C* in the third *R* strand, which blazed a new path at #2, #7, #10 for the recruitment of codons in Route1∼3, respectively (Figure 1a); (3) substitution of (+)GC∗C by (++)GC∗T, with the transition from *C* to *T* in the third *R* strand at #6, #19, #12 (Figure 1a and Figure 2), by which the remaining codons in Route1∼3 were recruited (Figure 1a). Thus, all the 64 codons have been recruited following the roadmap (Figure 1a, Figure 3a and Figure 4b).

According to the base substitutions on the roadmap, the recruitment order of the codon pairs from #1 to #32 is as follows (Figure 1a):

#1GGG·CCC, #2GGC·GCC, #3GGA·UCC, #4GAG·CUC, #5GAC·GUC, #6GGU·ACC, #7GCG·CGC, #8AGC·GCU, #9GCA·UGC, #10CGG·CCG, #11AGG·CCU, #12UGG·CCA, #13CGA·UCG, #14AGA·UCU, #15UGA·UCA, #16ACG·CGU, #17AGU·ACU, #18ACA·UGU, #19GUG·CAC, #20CAG·CUG, #21GAU·AUC, #22AUG·CAU, #23GAA·UUC, #24GUA·UAC, #25UAG·CUA, #26AAC·GUU, #27AAG·CUU, #28CAA·UUG, #29AUA·UAU, #30AAU·AUU, #31UAA·UUA, #32AAA·UUU;

and the recruitment order of the amino acids from No.1 to No.20 is as follows (Figure 1a):

No.1Gly, No.2Ala, No.3Glu, No.4Asp, No.5Val, No.6Pro, No.7Ser, No.8Leu, No.9Thr, No.10Arg, No.11Cys, No.12Trp, No.13His, No.14Gln, No.15Ile, No.16Met, No.17Phe, No.18Tyr, No.19Asn, No.20Lys.

The evolution of the genetic code can be divided into three stages (Figure 1a): the initiation stage (#1∼#6), the midway stage (#7∼#20, #24∼#27), and the ending stage (#21∼#23, #28∼#32). All the amino acids recruited in the initiation stage belong to phase I. The recruitment of amino acids along the roadmap is described step by step hereinafter, and the pair connections and route dualities on the roadmap will be explained according to the evolution of tRNAs and aaRSs in the following.

**Initiation**
*step 1*: **1Gly**Vacant #1
*step 2*: 1Gly Vacant #1 1Gly Vacant #2
*step 3*: 1Gly Vacant #1 1Gly **2Ala** #2
*step 4*: 1Gly Vacant #1 1Gly 2Ala #2 1Gly Vacant #3
*step 5*: 1Gly Vacant #1 1Gly 2Ala #2 1Gly Vacant #3 **3Glu** Vacant #4
*step 6*: 1Gly Vacant #1 1Gly 2Ala #2 1Gly Vacant #3 3Glu Vacant #4 **4Asp** Vacant #5
*step 7*: 1Gly Vacant #1 1Gly 2Ala #2 1Gly Vacant #3 3Glu Vacant #4 4Asp **5Val** #5
*step 8*: 1Gly **6Pro** #1 1Gly 2Ala #2 1Gly Vacant #3 3Glu Vacant #4 4Asp 5Val #5
*step 9*: 1Gly 6Pro #1 1Gly 2Ala #2 1Gly **7Ser** #3 3Glu Vacant #4 4Asp 5Val #5
*step 10*: 1Gly 6Pro #1 1Gly 2Ala #2 1Gly 7Ser #3 3Glu **8Leu** #4 4Asp 5Val #5
*step 11*: 1Gly 6Pro #1 1Gly 2Ala #2 1Gly 7Ser #3 3Glu 8Leu #4 4Asp 5Val #5 1Gly Vacant #6
*step 12*: 1Gly 6Pro #1 1Gly 2Ala #2 1Gly 7Ser #3 3Glu 8Leu #4 4Asp 5Val #5 1Gly **9Thr** #6
**Midway & ending**
*step 13:* (#1 ∼ #6 are fully filled by 1Gly to 9Thr, the same below for the following steps) 2Ala **10Arg** #7
and *the following steps* (omitting the previously fully filled #1 ∼ #(n-1) codon pairs in step #n, from #8 to #32):
  7Ser 2Ala #8; 2Ala **11Cys** #9; 10Arg 6Pro #10; 10Arg 6Pro #11; **12Trp** 6Pro #12; 10Arg 7Ser #13; 10Arg 7Ser #14; **stop** 7Ser #15; 9Thr 10Arg #16; 7Ser 9Thr #17; 9Thr 11Cys #18; 5Val **13His** #19; **14Gln** 8Leu #20; 4Asp **15Ile** #21; **16Met** 13His #22; 3Glu **17Phe** #23; 5Val **18Tyr** #24; stop 8Leu #25; **19Asn** 5Val #26; **20Lys** 8Leu #27; 14Gln 8Leu #28; 15Ile 18Tyr #29; 19Asn 15Ile #30; stop 8Leu #31; 20Lys 17Phe #32.

#### 2.2.2. Initiation

In the beginning, there was an *R* (*R* denotes purine) single-stranded DNA PolyG (Figure 1a,b, #1). By complementary base pairing formed a YR (*Y* denotes pyrimidine) double-stranded DNA PolyC·PolyG. Furthermore, by triplex base pairing CG∗G formed a YR∗R1 triple-stranded DNA PolyC·PolyG∗PolyG (Figure 1a,b, #1). The third R1 strand PolyG separated out of this YR∗R1 triple-stranded DNA, which then formed a new Y1R1 double-stranded DNA PolyC·PolyG. So far, there was only initial codon pair GGG·CCC (Figure 1a,b, #1).

In the initiation stage of the roadmap, the codon pairs from #1 to #6 were recruited along the roadmap, which constituted the initial subset of the genetic code:

#1GGG(1Gly)·CCC(6Pro), #2GGC(1Gly)·GCC(2Ala), #3GGA(1Gly)·UCC(7Ser),

#4GAG(3Glu)·CUC(8Leu), #5GAC(4Asp)·GUC(5Val), #6GGU(1Gly)·ACC(9Thr).

And in this stage were recruited the earliest 9 amino acids in order: 1Gly, 2Ala, 3Glu, 4Asp, 5Val, 6Pro, 7Ser, 8Leu, 9Thr, all of which belong to phase I amino acids [7,8]. For example, at codon pair position #6 on the roadmap, 1Gly and 9Thr are encoded by the codon pair $5^{\prime }GGT3^{\prime }$ in R6 strand and $5^{\prime }ACC3^{\prime }$ in Y6 strand, respectively. Although the initial subset is concise, two essential features of the roadmap, pair connection and route duality, had taken shape in this initiation stage (Figure 1a and Figure 3a).

Pair connection is an essential feature of the roadmap. A connected codon pair on the roadmap generally encode a common amino acid (Figure 1a and Figure 3b). For instance, the pair connection #1−Gly−#2 indicates that both GGG in #1 and GGC in #2 encode the common amino acid Gly. Pair connections reveal the close relationship between recruitment of codons and recruitment of amino acids, which will be explained later according to the evolution of tRNAs.

Route duality is another essential feature of the roadmap, which shows the relationship of pair connections between different routes (Figure 1a and Figure 3b). For instance, the route duality

#1−Gly−#3∼#2−Gly−#6

indicates that the pair connection #1−Gly−#3 in Route0 and the pair connection #2−Gly−#6 in Route1 are dual, which encode a common amino acid Gly. Route dualities generally exist between Route0 and Route3, or between Route1 and Route2 (Figure 3b), which will be explained later according to the evolution of aaRSs.

Glycine, the simplest amino acid, is encoded by the cytosine triplet, the simplest nitrogen base. Glycine has been identified in the coma of comet [52] and could be the first amino acid on earth. Here, glycine Gly is also the first amino acid recruited on the roadmap. In the initiation stage of the roadmap, the non-chiral Gly helped to create the first pair connection #1−Gly−#2, recruiting chiral Ala at #2 (Figure 1a). Furthermore, the non-chiral Gly also helped to create the first route duality on the roadmap (Figure 1a):

#1−Gly−#3∼#2−Gly−#6.

This route duality played a central role in the initiation stage; consequently, the initial subset played a central role in the midway stage (Figure 3a). The chirality was required at the beginning of the roadmap by the triplex DNA itself (Figure 1a,b). Even so, there was still a transition period from non-chirality to chirality, in consideration of the special role of non-chiral Gly. Competition between opposite homochiral roadmap systems resulted in the homochirality by a winner-take-all game [53].

#### 2.2.3. Midway

The genetic codes evolved along four routes Route0−3, respectively, where 8 codon pairs in each route evolved in the order of four hierarchies Hierarchy1∼4, respectively (Figure 1a). The roadmap can be divided into two groups: the early hierarchies Hierarchy1∼2 and the late hierarchies Hierarchy3∼4. It can also be divided into two groups: the initial route Route0 (all-purine codons pairing with all-pyrimidine codons) and the expanded routes Route1∼3 (purine-pyrimidine-mixing codons).

In the midway stage of the roadmap, the genetic codes expanded spontaneously from the initial subset (Figure 1a and Figure 3a). Each of the 6 codon pairs in the initial subset expanded to three additional codon pairs, respectively, by route dualities. Details are as follows. The codon pair #2 in the initial subset expanded to the three continual codon pairs #7, #8 and #9 by route duality

#2−Ala−#8∼#7−Ala−#9;

the codon pair #1 in the initial subset expanded to the three continual codon pairs #10, #11, and #12 by route duality

#1−Pro−#11∼#10−Pro−#12;

the codon pair #3 in the initial subset expanded to the three continual codon pairs #13, #14, and #15 by route duality

#3−Ser−#14∼#13−Ser−#15;

the codon pair #6 in the initial subset expanded to the three continual codon pairs #16, #17, and #18 by route duality

#6−Thr−#17∼#16−Thr−#18;

the codon pair #5 in the initial subset expanded to the three codon pairs #19, #24, and #26 by route duality

#5−Val−#26∼#19−Val−#24;

and the codon pair #4 in the initial subset expanded to the three codon pairs #20, #25, and #27 by route duality

#4−Leu−#27∼#20−Leu−#25.

The recruitment order of the codon pairs and the recruitment order of the amino acids are intricately well organised and coherent, according to the subtle roadmap (Figure 1a and Figure 3a). In the initiation stage, firstly, the amino acid No.1 was recruited with the codon pair #1, remaining a vacant position. Subsequently, No.1 and No.2 were recruited with the codon pair #2; No.1 was recruited with the codon pair #3, remaining a vacant position; No.3 was recruited with the codon pair #4, remaining a vacant position; No.4 and No.5 were recruited with the codon pair #5; No.6 filled up the vacant position of #1; No.7 filled up the vacant position of #3; No.8 filled up the vacant position of #4; No.1 and No.9 were recruited with the codon pair #6 (Figure 3a). Thus, the framework of the genetic code had been established at the end of the initiation stage. From #7 on, the latecomer amino acids no longer jumped the queue in recruitment so that there were no more vacant positions in the recruited codon pairs. Details are as follows. No.2 and No.10 amino acids were recruited with the codon pair #7; and, subsequently, No.2 and No.7 were recruited with #8; No.2 and No.11 were recruited with #9; No.6 and No.10 were recruited with #10; No.6 and No.10 were recruited with #11; No.6 and No.12 were recruited with #12; No.7 and No.10 were recruited with #13; No.7 and No.10 were recruited with #14; No.7 and stop were recruited with #15; No.9 and No.10 were recruited with #16; No.7 and No.9 were recruited with #17; No.9 and No.11 were recruited with #18; No.5 and No.13 were recruited with #19; No.8 and No.14 were recruited with #20; No.4 and No.15 were recruited with #21; No.13 and No.16 were recruited with #22; No.3 and No.17 were recruited with #23; No.5 and No.18 were recruited with #24; No.8 and stop were recruited with #25; No.5 and No.19 were recruited with #26; No.8 and No.20 were recruited with #27; No.8 and No.14 were recruited with #28; No.15 and No.18 were recruited with #29; No.15 and No.19 were recruited with #30; No.8 and stop were recruited with #31; No.17 and No.20 were recruited with #32 (Figure 3a).

Take, for example, from #1 to #29, the evolution of the genetic code along the roadmap can be described in details as follows (Figure 1a,b and Supplementary Movie S1). Starting from the position #1 (Figure 1b, #1), an *R* single-stranded DNA brought about a YR double-stranded DNA; next, the YR double-stranded DNA brought about a YR∗R1 triple-stranded DNA (the number 1 denotes #1, similar below); next, an R1 single-stranded DNA departed from the YR∗R1 triple-stranded DNA; next, the R1 single-stranded DNA brought about a R1Y1 double-stranded DNA. Thus, the codon pair GGG·CCC were achieved at #1. At the beginning of #7 (Figure 1b, #7), the R1Y1 double-stranded DNA was renamed as Y1R1 double-stranded DNA, where the $180^{\circ }$ rotation in writing did not change the right-handed helix; next, the Y1R1 double-stranded DNA brought about a Y1R1∗R7 triple-stranded DNA, through the transversion from *G* to *C*, where the stability (+) of CG∗G increased to the stability (4+) of CG∗C; next, an R7 single-stranded DNA departed from the Y1R1∗R7 triple-stranded DNA; next, the R7 single-stranded DNA brought about a R7Y7 double-stranded DNA. Thus, the codon pair GCG·CGC were achieved at #7. The case of #19 is similar to #7 (Figure 1b, #19); the codon pair GTG·CAC were achieved through the transition from *C* to *T*, where the stability (+) of GC∗C increased to the stability (2+) of GC∗T. The case of #24 is also similar to #7 (Figure 1b, #24); the codon pair GTA·TAC were achieved through the common transition from *G* to *A*, where the stability (+) of CG∗G increased to the stability (2+) of CG∗A. At the position #29 (Figure 1b, #29), the codon pair GCG·CGC in Y24R24 are non-palindromic in consideration that both GCG and CGC do not read the same backwards as forwards. In this case, a reverse operation is necessary so that the obtained codon pair CAT·ATG in y24r24 read reversely the same as the codon pair TAC·GTA in Y24R24. The process from y24r24 to R29Y29 is still similar to the case of #7; the codon pair ATA·TAT were achieved through the transition from *G* to *A*, where the stability (+) of CG∗G increased to the stability (2+) of CG∗A. Other processes on the roadmap are similar to the above example (Figure 1a,b). The reverse operation is unnecessary in the cases of #2, #7, #10, #11, #3, #4, #16, #9, #19, #27, #23, #22, #24 after palindromic codon pairs and the last one #32 (Figure 1a), whereas the reverse operation is necessary in the remaining cases of #5, #6, #8, #12, #13, #14, #15, #17, #18, #20, #21, #25, #26, #28, #29, #30, #31 (Figure 1a).

#### 2.2.4. The Ending

So far, the genetic code table had been expanded from the 6 codon pairs in the initial subset to the 6+18 codon pairs by route duality; the remaining 8 codon pairs were recruited into the genetic code table in the ending stage of the roadmap (Figure 1a and Figure 3a). There were 2 codon pairs remained in each of the four routes Route0−3, respectively. They satisfied pair connections as follows: #23−Phe−#32, #21−Ile−#30, #22−Met/Ile−#29, #28−Leu−#31 (Figure 3a). Two of them satisfied route duality (Figure 3a):

#21−Ile−#30∼#22−Met/Ile−#29.

The last two stop codons appeared in the pair connection #25−stop−#31 (Figure 1a and Figure 3a). When the last two amino acids were recruited through the base pairs #26−Asn−#30 and #27−Lys−#32, the codon UAG at #25 had to be selected as a stop codon. The codon UAA at #31 was selected as the last stop codon, due to lack of corresponding tRNA.

The non-standard codons also satisfy codon pairs and route dualities on the roadmap (Figure 1a). The codon pairs pertaining to non-standard codons are as follows: #11−Arg(Ser,stop)−#14, #4−Leu(Thr)−#27 in Route0; none in Route1; #22−(Met)−#29 in Route2; #20−Leu(Thr,Gln)−#25, #12−(Trp)−#15, #25−stop(Gln)/Leu−#31, #28−Leu(Gln)−#31 in Route3. Majority of non-standard codons appear in the last Route3 (Figure 1a). Route dualities of non-standard codons exist between Route0 and Route3 (Figure 1a):

$$\begin{array}{ccc} \#4-Leu\;(Thr)-\#27 & ∼ & \#20-Leu\;(Thr)-\#25\\ \#11-(stop)-\#14 & ∼ & \#12-Trp/stop-\#15, \end{array}$$

where the first stop codon UGA at #15 is dual to the non-standard stop codons in Route0.

The choice of the genetic code was by no means random, which resulted from the increasing stabilities of triplex base pairs in the substitutions [10,11], where the rotation of the single glycosidic bond between base and deoxiribose has been considered in the opposite direction. It had been emphasised that the roadmap followed the strict rule that the stabilities of triplex base pairs monotonically increase (Figure 2). Note that the roadmap had tried its best to avoid the unstable triplex DNA. The roadmap (Figure 1a) is the only possible one that has avoided the unstable triplex base pairs (−) GC∗A, AT∗C and AT∗A, as shown in Table 1, while other eliminated possible roadmaps cannot avoid.

Among the 16 possible triplex base pairs, there are three relatively unstable triplex base pairs. So, the statistical ratio of instability for the triplex base pairs is 3/16. However, the ratio of instability for the triplex base pairs on the roadmap is much smaller. There are 49 triplex DNAs through #1 to #32 on the roadmap, which involve 3×49=147 triplex base pairs (Figure 1a). The relatively unstable triplex base pairs GC∗A and AT∗C have not appeared on the roadmap; only the relatively unstable triplex base pair AT∗A has appeared inevitably for 7 times in the reverse operations so as to fulfil all the permutations of 64 codons (Figure 1a). The ratio of instability 7/147 on the roadmap is much smaller than the ratio of instability 3/16 by the statistical requirement. When the relatively unstable AT∗A appears at the positions #15, #17, #21, #25, #29, #30, and #31, both stabilities of the other two triplex base pairs in the triplex DNA are (4+) (Figure 1a), which compensates the instability of the triplex DNA to some extent. The amino acid Ile, whose degeneracy uniquely is three, occupied three positions #21, #29, and #30 among those 7 positions. In addition, the three stop codons occupied other three neighbour positions #15, #25 and #31 (Figure 1a). The first stop codon UGA appeared at the position #15, where the relatively unstable AT∗A appeared firstly (Figure 1a). According to the primordial translation mechanism, the weak combination of AT∗A might help to assign stop codons. The route dualities played significant roles in the midway stage, where the remnant codons were chosen as the stop codons (Figure 1a and Figure 3a). The stop codon appeared as early as the midway of the evolution of the genetic code (Figure 1a and Figure 3a), which indicates that the genetic code had been taken shape around the midway to promote the formation of the primitive life. Not until the fulfilment of the genetic code did the translation efficiency increase notably by recognising all the 64 codons.

### 2.3. Origin of tRNA

**Figure 5:**
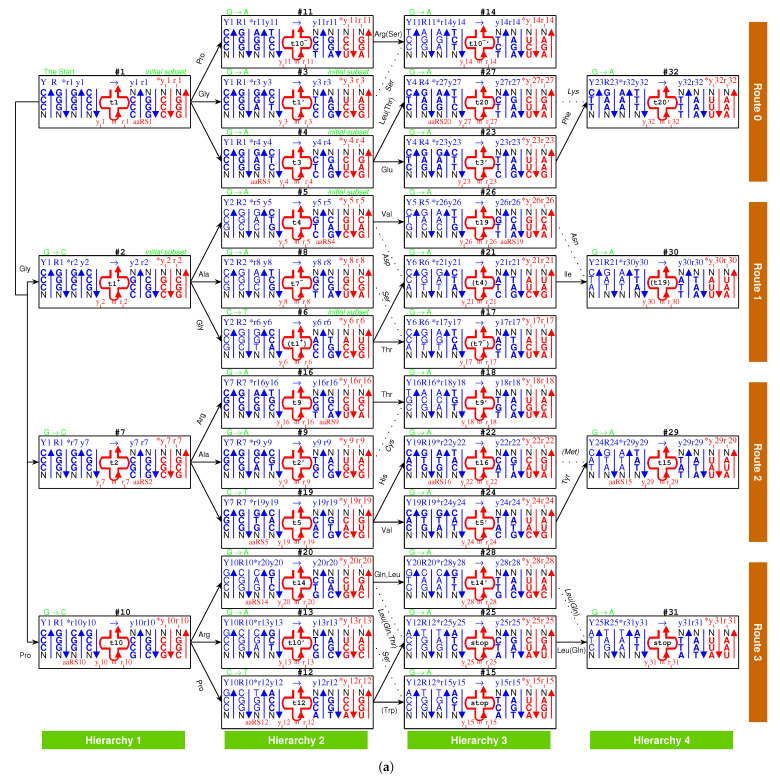

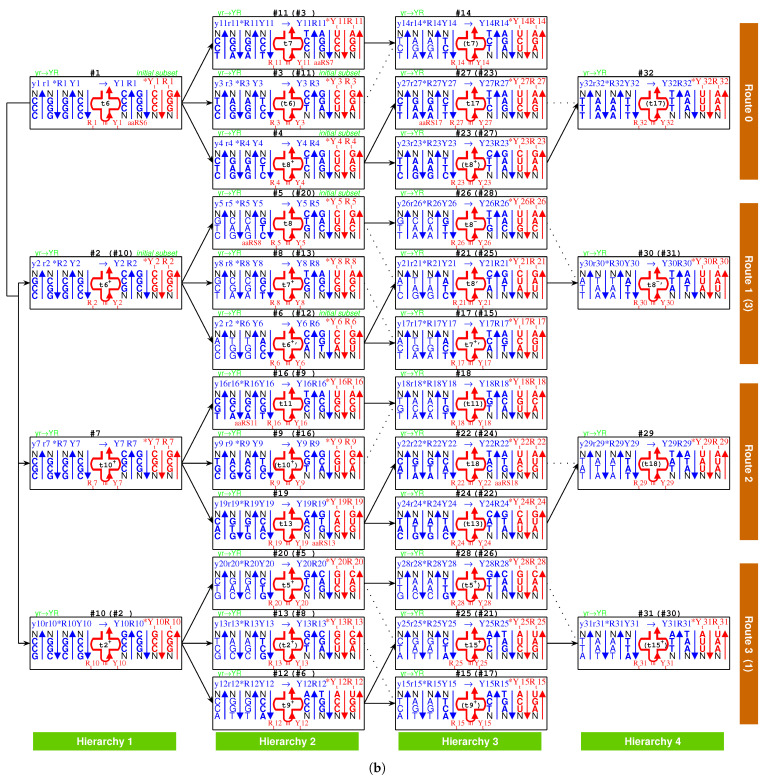

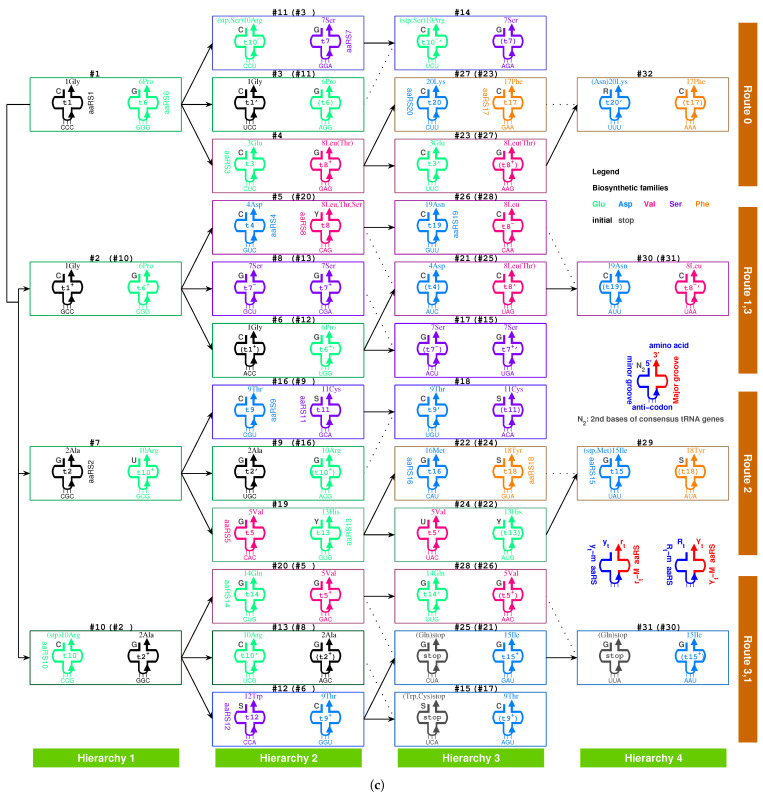
The origin and evolution of tRNAs along the roadmap. (**a**) The evolution of the $5^{\prime }y_{t}r_{t}3^{\prime }$ type tRNAs by the triplex base pairings $yr*y_{t}$ and $yr*r_{t}$. (**b**) The evolution of the $5^{\prime }R_{t}Y_{t}3^{\prime }$ type tRNAs by the triplex base pairings yr∗R, yr∗Y and $YR*Y_{t}$ and $YR*R_{t}$. The node numbers #n on the roadmap may exchange within or between routes because the sequences of *Y* and *R* are reverse to the sequences of *y* and *r*, respectively. (**c**) The coevolution of tRNAs with aaRSs along the roadmap, which determines the pair connections and route dualities. The aaRSs aaRS1 to aaRS20 combine, respectively, with the tRNAs t1 to t20 from certain major/minor groove side. The complementary relationship between the pyrimidine $y_{t}$ strand of the $5^{\prime }y_{t}r_{t}3^{\prime }$ type tRNAs and the purine $R_{t}$ strand of the $5^{\prime }R_{t}Y_{t}3^{\prime }$ type tRNAs agrees with the complementary relationship between *G* and *C* for the second bases of the consensus genes of tRNAs, especially for the early tRNAs in Route0 and in Hierarchy1.

**Figure 6:**
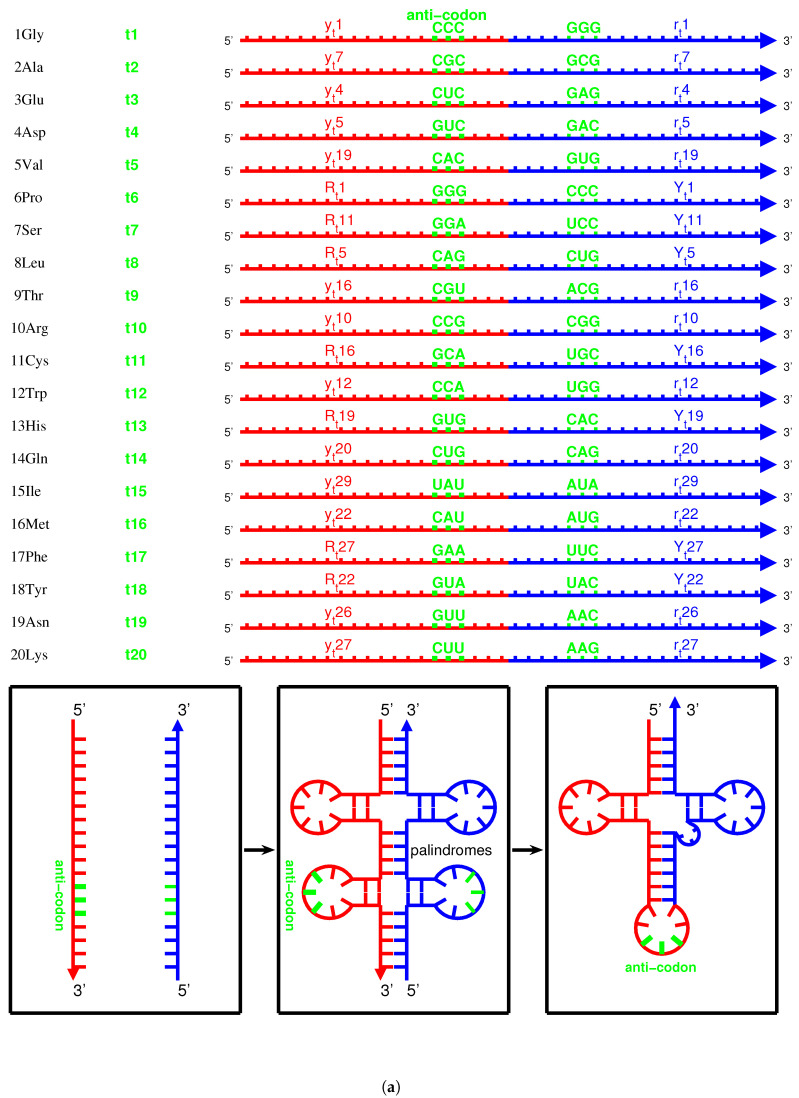

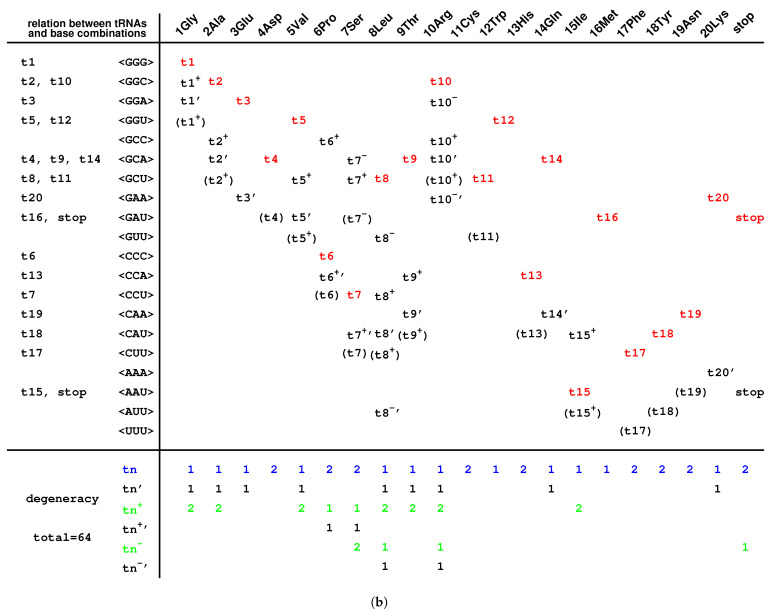
(**a**) The assembly of tRNAs. The tRNAs t1-t20 with anti-codons (Figure 5c) are listed here to carry the amino acids from No.1 to No.20, respectively. The two complimentary single-stranded RNAs for each tRNA join together and fold into a cloverleaf shape by taking advantage of the complementarity between the two strands. The joining position of the two strands is near to the 3${}^{\prime }$ side of the anti-codon loop, which agrees with the position of introns in tRNA genes in observations. The anti-codons situate in the $3^{\prime }$-ends of the $y_{t}$ strand or $R_{t}$ strand. The palindromic sequences tend to form loops of the tRNAs. The para-codon of tRNA are non-palindromic or palindromic which adapt to the aaRSs (Figure 7 and Figure 8). (**b**) The cognate tRNAs. Explanation of the number of canonical amino acids as 20 based on the relationship between the types of cognate tRNAs and the 20 types of base combinations. The primer tRNAs generally appeared earlier than the derivative tRNAs. The primer tRNAs generally distribute along the diagonal line due to the chronological arrangements for both the 20 amino acids and the 20 base combinations, considering the substitution order *G*, *C*, *A*, *U* along the roadmap. The codon degeneracies 6, 4, 3, 2, and 1 are due to the tRNA evolution from tn to $tn^{+}$ and $tn^{-}$, as well as from tn to $tn^{\prime }$, etc., all of which can be recognised by the corresponding aaRSn.

**Figure 7:**
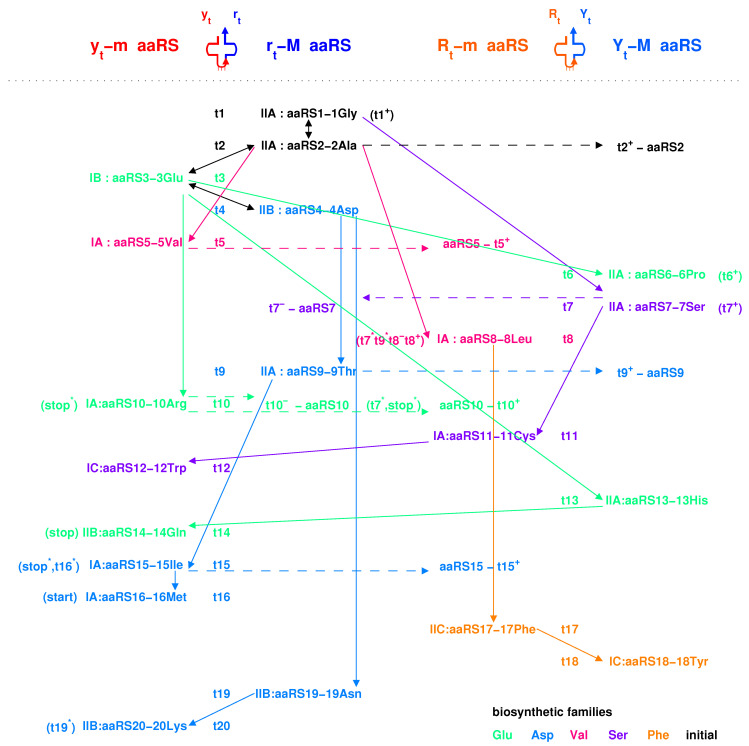
The coevolution of tRNAs with aaRSs. The coevolution of the four classes of aaRSs and the corresponding two types of tRNAs in accordance with the biosynthetic families indicated in certain colours. The ancestor of aaRS, namely aaRS1, corresponding to the non-chiral amino acid 1Gly, belongs to the $r_{t}-M$ class. The codon degeneracy are due to the coevolution of tRNAs with aaRSs, where the surplus tRNAs were chosen by the rare aaRSs. There are some truths in the traditional classifications of aaRSs, but the evolutionary relationships of aaRSs are so intricate, as shown here. The start and stop codons generally appear in the positions corresponding to $y_{t}-m$ class. The non-standard codons also evolved as alternative choices of tRNAs by aaRSs.

The roadmap illustrates the coevolution of the genetic code with the amino acids, where tRNAs and aaRSs play an intermediary role. The expansion of the genetic code along the roadmap can be explained by the coevolution of tRNAs with aaRSs (Figure 5c, Figure 6b and Figure 7). The cloverleaf shape of tRNA can be explained by assembling the two complementary RNA strands separated from triplex nucleic acid D·D∗R in the triplex picture (Figure 6a). The origin of aaRS will be explained next.

#### 2.3.1. Anti-Codon

When studying the evolution of the genetic code, we were focused on only three bases in the triplex DNA. However, when studying the origin of tRNAs, it is necessary to study the evolution of entire sequences of both triplex DNA and triplex nucleic acid D·D∗R, where the third RNA strands in D·D∗R can be used to assemble tRNAs (Figure 5a,b and Figure 6a). According to the order of the relative stabilities of YR∗Y for the 8 kinds of triplex nucleic acids: D·D∗D, D·D∗R, R·D∗R, R·D∗D>D·R∗R, R·R∗R>>R·R∗D, D·R∗D [50,54], the relative stabilities of D·D∗D and D·D∗R are greater than the relative stabilities of other kinds of triplex nucleic acids. The choice of triplex DNA for the roadmap and the choice of D·D∗R for the origin of tRNAs are based on the observed relative stabilities. And the other kinds of triplex nucleic acids can be neglected due to their less probabilities to appear.

There are four types of RNA strands for assembling tRNAs that were generated by the triplex base pairing of triplex nucleic acids D·D∗R: via the triplex nucleic acid $yr*y_{t}$, via the triplex nucleic acid $yr*r_{t}$ (Figure 5a,c), and via the triplex nucleic acid $YR*Y_{t}$, via the triplex nucleic acid $YR*R_{t}$ (Figure 5b,c), where the subscript *t* indicates that theses RNA strands $y_{t}$, $r_{t}$ and $Y_{t}$, $R_{t}$ are used to assemble tRNA (Figure 5a,b and Figure 6a). The sequences $Y_{t}$, $R_{t}$ are the respective reverse sequences of $y_{t}$ and $r_{t}$. There is a difference in the sequence evolution along the roadmap between purine strands and pyrimidine strands. The pyrimidine sequences $Y_{t}$, $y_{t}$ and the purine sequences $R_{t}$, $r_{t}$ are complementary, respectively, owing to the triplex pairing with the purine DNA strand and the pyrimidine DNA stand in the triplex nucleic acids D·D∗R, respectively. These tRNA strands coevolved with the triplex DNA along the roadmap. Therefore, the evolution of the anti-codons on tRNAs can be explained according to the evolution of the genetic code along the roadmap. The evolution of aaRSs should be considered next. After separating from the triplex nucleic acids D·D∗R, the pair of complementary single RNA strands $y_{t}$ and $r_{t}$, or $R_{t}$ and $Y_{t}$, can concatenate and fold into a cloverleaf-shaped tRNA [55,56,57,58,59], whose anti-codon corresponds to the codon of the triplex DNA on the roadmap (Figure 6a). Owing to the different positions of anti-codons in the RNA strands, either near to $3^{\prime }$-ends or near to $5^{\prime }$-ends, it must be seriously considered for the different reading directions between $Y_{t}$, $R_{t}$ and $y_{t}$, $r_{t}$ (Figure 6a). There were two types of tRNAs: the type $5^{\prime }y_{t}r_{t}3^{\prime }$ tRNA and the type $5^{\prime }R_{t}Y_{t}3^{\prime }$ tRNA (Figure 5a,b), where the anti-codons are near to the $3^{\prime }$-end of the RNA strand $y_{t}$ and the $3^{\prime }$-end of the RNA strand $R_{t}$, respectively. The other concatenated RNA strands $5^{\prime }r_{t}y_{t}3^{\prime }$ and $5^{\prime }Y_{t}R_{t}3^{\prime }$ cannot evolve together with the above two types of tRNAs because the corresponding triplets would be on the acceptor arms rather than on the anti-codon loops.

It is possible to explain the sequence evolution of tRNAs in detail along the roadmap (Figure 5a–c and Figure 6a). For example, the tRNA t2 for 2Ala can form by concatenating $y_{t}7$ and $r_{t}7$, which are generated by triplex base parings $y7r7*y_{t}7$ and $y7r7*r_{t}7$ at the branch node #7. The anti-codon CGC near the $3^{\prime }$-end of the strand $y_{t}7$ is palindromic. The two complementary strands $y_{t}7$ and $r_{t}7$ can combine into a cloverleaf-shaped type $5^{\prime }y_{t}r_{t}3^{\prime }$ tRNA t2 by concatenating, pairing, and folding (Figure 6a). Thus, anti-codon arm of t2 contains the anti-codon CGC, which corresponds to Ala, with the help of aaRS; consequently, the codon GCG at the *R* DNA strand in #7 is assigned to Ala. The sequences evolve from #7 to #16 along the roadmap. As another example, the codons at the position #16 is non-palindromic, where the type $5^{\prime }y_{t}r_{t}3^{\prime }$ tRNA t9 and the type $5^{\prime }R_{t}Y_{t}3^{\prime }$ tRNA t11 are assembled by concatenating $y_{t}16$ and $r_{t}16$ for t9 and by concatenating $R_{t}16$ and $Y_{t}16$ for t11, respectively (Figure 6a). Hence, the codon ACG at #16 and the reversely complimentary codon UGC at #9 are assigned to 9Thr and 11Cys, respectively.

There are 4 pairs of palindromic codons: #1CCC·GGG, #4CUC·GAG, #7CGC·GCG, #19CAC·GUG in the 16 branch nodes of the roadmap (Figure 1a). Accordingly there are 12 non-palindromic codons among the branch nodes at the positions #2, #5, #6, #10, #11, #12, #16, #20, #21, #23, #24, and #25. The sets of complementary pairs of RNA strands are the same for the two routes because of the bijection between Route1 and Route3 in the sense of reverse relationship (Figure 1a). Thus, there are totally 4+(12−4)×2=20 pairs of complementary single RNA strands (4 palindromic codons, and the 12 non-palindromic codons minus 4 identities between Route1 and Route3), which can assemble into 20 groups of cognate tRNAs, respectively. This could be among the reasons why there are 20 canonical amino acids.

There is another reason at the sequence level for the number “20” of the canonical amino acids (Figure 6b). There are 64 triple permutations for the 4 bases, which accounts for the number 64 of the codons. However, little attention has been paid to the 20 triple combinations for the 4 bases. The products p(i)∗p(j)∗p(k) (i,j,k=G,C,A,T) are the same, respectively, for the 20 groups of combinations for the 4 bases (Figure 6b), owing to the multiplication exchange law, where p(i) denotes the base compositions for i=G,C,A,T. The products determine the average interval distances of codons in genome sequences. Therefore, there are 20 classes of genomic codon interval distributions according to the 20 combinations rather than the 64 permutations of the 4 bases [53]. Consequently, there are 20 cognate tRNA-synthetase systems so as to improve the translation efficiency for tRNAs to recognise the corresponding codons, considering the 20 average interval distances of codons. So, the number “20” of the canonical amino acids actually should be attributed to a statistical origin at the sequence level. The 20 combinations of the 4 bases can be divided into 4 groups: <G>, <C>, <A>, <T>. Hierarchy1 and Hierarchy2 correspond <G> and <C>; Hierarchy3 and Hierarchy4 correspond to <A> and <T>. Their positions on the roadmap are Hierarchy1∼2Y:<G>, Hierarchy1∼2R:<C>, Hierarchy3∼4Y:<A>, Hierarchy3∼4R:<T>. Each group can be divided into 5 combinations, which correspond to Route0 or Route1∼3, respectively. In the case <G>, <G,G,G> and <G,G,A> belong to Route0; <G,G,C>, <G,G,T>, and <G,C,A> belong to Route1∼3, and it is similar for the other cases <C>, <A>, <T>. These 20 combinations roughly correspond to the 20 cognate tRNAs (Figure 6b). This rough correspondence shows that the codons, especially those in Hierarchy1∼3, are assigned to the tRNAs based on the combinations, considering that the codons in Hierarchy4 are AT-rich, and the context sequences tend to form AT-rich repeats. Concretely speaking, the group of codons in the combinations <GGG>, <GGC>, <GGA>, <GGU>, <GCA>, <GCU>, <GAA>, <GAU>, <CCC>, <CCA>, <CCU>, <CAA>, <CAU>, <CUU>, <AAU> are assigned, respectively, to t1, t2 and t10, t3, t5 and t12, t4 and t9 and t14, t8 and t11, t20, t16, t6, t13, t7, t19, t18, t17, t15 (Figure 6b). In addition, the first stop codon appeared halfway in the evolution of tRNAs (Figure 6b). The order of combinations are simply organised by the bases in the order “*G*”, “*C*”, “*A*”, “*U*” (Figure 6b), considering the substitutions “*G* to *C*”, “*G* to *A*”, “*C* to *U*” on the roadmap (Figure 1a). And the amino acids are in the recruitment order. Then, a rough diagonal distribution of tRNAs has been obtained (Figure 6b), which is due to the evolutionary relationship between the genetic code and amino acids.

#### 2.3.2. Evolution of tRNA

There was a post-initiation-stage stagnation (Figure 1a) between the initiation stage and the midway stage of the roadmap. Such a stagnation in the prebiotic evolution was just to await the birth of functional macromolecules. In this period, oligonucleotides with arbitrary finite sequences can be generated via the base substitutions *G* to *A*, *G* to *C*, and *C* to *T* in the triplex picture. The primordial sequences of the prototype tRNAs and the template RNAs of prototype aaRSs can be generated along the roadmap. In the light of complicated interactions between oligonucleotides and amino acids, some early tRNAs with certain anti-codons can be generated in the sequence evolution along the roadmap so as to carry the corresponding prebiotically synthetised phase I amino acids, respectively. These tRNAs were not necessarily homologous, as long as they were capable of fulfilling their respective tasks. There are two independent codon systems for tRNAs: the anti-codons and the para-codons. The anti-codons evolved along the roadmap, while the para-codons evolved with aaRSs (Figure 5c and Figure 7). When the para-codons did not evolve but the anti-codons evolved, only cognate tRNAs originated. However, when both the para-codons and the anti-codons evolved, more new tRNAs originated to carry the remaining amino acids.

There exists an assignment scheme for the genetic code. The 64 codons can be assigned to the 20 amino acids and stop codons with the help of approximate four dozens of tRNAs: t1, $t1^{\prime }$, $t1^{+}$, t2, $t2^{\prime }$, $t2^{+}$, t3, $t3^{\prime }$, t4, t5, $t5^{\prime }$, $t5^{+}$, t6, $t6^{+}$, $t6^{+}{}^{\prime }$, t7, $t7^{+}$, $t7^{-}$, $t7^{-}{}^{\prime }$, t8, $t8^{\prime }$, $t8^{+}$, $t8^{-}$, $t8^{-}{}^{\prime }$, t9, $t9^{\prime }$, $t9^{+}$, t10, $t10^{\prime }$, $t10^{+}$, $t10^{-}$, $t10^{-}{}^{\prime }$, t11, t12, t13, t14, $t14^{\prime }$, t15, $t15^{+}$, t16, t17, t18, t19, t20, $t20^{\prime }$ (Figure 5c and Figure 6b). The naming rules for tRNAs are as follows. The tRNA series numbers are named after the recruitment order of the respective canonical amino acids. The prime tRNAs t1∼t20 are the early recruited tRNAs that coevolve with the corresponding aaRSs. The derivative tRNAs $tn^{+}$ are the cognate tRNAs expanded within the codon boxes, namely with the same first two bases in codons. The derivative tRNAs $tn^{-}$ are the cognate tRNAs expanded outside the codon boxes. The derivative tRNAs $tn{}^{\prime }$, $n^{+}{}^{\prime }$ and $tn^{-}{}^{\prime }$ are the cognate tRNAs needed by wobble pairing rules. The bracket in “(tn)” indicates the same tRNA tn. It is also possible to generate more or less new tRNAs in the triplex picture for different species, so the numbers of tRNAs are different among species.

On one side, the tRNAs can recognise the respective codons according to the genetic code evolution along the roadmap. On the other side, they can recognise the respective aaRSs to combine with the respective aminoacyls. Among the 20 prime tRNAs t1∼t20, there are 13 type $5{}^{\prime }y_{t}r_{t}3{}^{\prime }$ tRNAs (t1, t2, t3, t4, t5, t9, t10, t12, t14, t15, t16, t19, t20) and 7 type $5{}^{\prime }R_{t}Y_{t}3{}^{\prime }$ tRNAs (t6, t7, t8, t11, t13, t17, t18) (Figure 5c). The codons for the type $5{}^{\prime }y_{t}r_{t}3{}^{\prime }$ prime tRNAs are situated in the purine strand on the roadmap, whose first base are purine, except t10, t12, t14, while the codons for the type $5{}^{\prime }R_{t}Y_{t}3{}^{\prime }$ prime tRNAs are situated in the Y strand on the roadmap, whose first base are pyrimidine. In total, there are 6 prime tRNAs (t1, t3, t6, t7, t17, t20) in Route0, 3 prime tRNAs (t4, t8, t19) in Route1, 8 prime tRNAs (t2, t5, t9, t11, t13, t15, t16, t18) in Route2, and 3 prime tRNAs (t10, t12, t14) in Route3 (Figure 5c). The majority of prime tRNAs situated in the branch nodes, except t15, t17, t19, t20 (Figure 5c). For each amino acid, several cognate tRNAs can be generated at certain steps of the roadmap.
1GlyaaRS1(GlyRS)t1(GGG), $t1^{\prime }\left(GGA\right)$, $t1^{+}\left(GGC,GGU\right)$2AlaaaRS2(AlaRS)t2(GCG), $t2^{\prime }\left(GCA\right)$, $t2^{+}\left(GCC,GCU\right)$3GluaaRS3(GluRS)t3(GAG), $t3^{\prime }\left(GAA\right)$4AspaaRS4(AspRS)t4(GAC,GAU)5ValaaRS5(ValRS)t5(GUG), $t5^{\prime }\left(GUA\right)$, $t5^{+}\left(GUC,GUU\right)$6ProaaRS6(ProRS)t6(CCC,CCU), $t6^{+}\left(CCG\right)$, $t6^{+}{}^{\prime }\left(CCA\right)$7SeraaRS7(SerRS)t7(UCC,UCU), $t7^{+}\left(UCG\right)$, $t7^{+}{}^{\prime }\left(UCA\right)$, $t7^{-}\left(AGC,AGU\right)$8LeuaaRS8(LeuRS)t8(CUG), $t8^{\prime }\left(CUA\right)$, $t8^{+}\left(CUC,CUU\right)$, $t8^{-}\left(UUG\right)$, $t8^{-}{}^{\prime }\left(UUA\right)$9ThraaRS9(ThrRS)t9(ACG), $t9^{\prime }\left(ACA\right)$, $t9^{+}\left(ACC,ACU\right)$10ArgaaRS10(ArgRS)t10(CGG),$t10^{\prime }\left(CGA\right)$,$t10^{+}\left(CGC,CGU\right)$,$t10^{-}\left(AGG\right)$,$t10^{-}{}^{\prime }\left(AGA\right)$11CysaaRS11(CysRS)t11(UGC,UGU)12TrpaaRS12(TrpRS)t12(UGG)13HisaaRS13(HisRS)t13(CAC,CAU)14GlnaaRS14(GlnRS)t14(CAG), $t14^{\prime }\left(CAA\right)$15IleaaRS15(IleRS)t15(AUA),$t15^{+}\left(AUC,AUU\right)$16MetaaRS16(MetRS)t16(AUG)17PheaaRS17(PheRS)t17(UUC,UUU)18TyraaRS18(TyrRS)t18(UAC,UAU)19AsnaaRS19(AsnRS)t19(AAC,AAU)20LysaaRS20(LysRS)t20(AAG), $t20^{\prime }\left(AAA\right)$

The following evolution of derivative tRNAs can be explained by the base substitution *G* to *A* along the roadmap (Figure 5c): t1(GGG) to $t1^{\prime }\left(GGA\right)$, t2(GCG) to $t2^{\prime }\left(GCA\right)$, t3(GAG) to $t3^{\prime }\left(GAA\right)$, t5(GUG) to $t5^{\prime }\left(GUA\right)$, $t6^{+}\left(CCG\right)$ to $t6^{+}{}^{\prime }\left(CCA\right)$, $t7^{+}\left(UCG\right)$ to $t7^{+}{}^{\prime }\left(UCA\right)$, t8(CUG) to $t8^{\prime }\left(CUA\right)$, $t8^{-}\left(UUG\right)$ to $t8^{-}{}^{\prime }\left(UUA\right)$, t9(ACG) to $\;t9^{\prime }\left(ACA\right)$, t10(CGG) to $t10^{\prime }\left(CGA\right)$, $t10^{-}\left(AGG\right)$ to $t10^{-}{}^{\prime }\left(AGA\right)$, t14(CAG) to $t14^{\prime }\left(CAA\right)$, t20(AAG) to $t20^{\prime }\left(AAA\right)$. Moreover, the following evolution of derivative tRNAs can be explained by the base substitution *G* to *C* along the roadmap (Figure 5c): t1(GGG) to $t1^{+}\left(GGC,GGU\right)$, t2(GCG) to $t2^{+}\left(GCC,GCU\right)$, t5(GUG) to $t5^{+}\left(GUC,GUU\right)$, $t6^{+}\left(CCG\right)$ to t6(CCC,CCU), t8(CUG) to $t8^{+}\left(CUC,CUU\right)$, t9(ACG) to $t9^{+}\left(ACC,ACU\right)$, t10(CGG) to $t10^{+}\left(CGC,CGU\right)$. However, the following tRNAs can recognise the respective two codons whose third bases are *C* or *U*, owing to the wobble pairing (Figure 5c): $t1^{+}\left(GGC,GGU\right)$, $t2^{+}\left(GCC,GCU\right)$, t4(GAC,GAU), $\;\;t5^{+}\left(GUC,GUU\right)$, t6(CCC,CCU), t7(UCC,UCU), $\;\;t7^{-}\left(AGC,AGU\right)$, $t8^{+}\left(CUC,CUU\right)$, $t9^{+}\left(ACC,ACU\right)$, $t10^{+}\left(CGC,CGU\right)$, t11(UGC,UGU), t13(CAC,CAU), $t15^{+}\left(AUC,AUU\right)$, t17(UUC,UUU), t18(UAC,UAU), t19(AAC,AAU).

The wobble pairing rules can be explained by the origin and evolution of tRNAs in the triplex picture. The transition from *C* to *T* occurred at the position #6 on the roadmap, which resulted in the wobble pairing rule G:UorC. Taking y2r2 as a template, $y_{t}2$ with GCC is formed by the triplex base pairing, while $r_{t}2$ with GGC and $r_{t}^{\prime }2$ with GGU are formed, where the transition from *C* to *U* occurred in the formation of $r_{t}^{\prime }2$. The complementary strands $y_{t}2$ and $r_{t}^{\prime }2$ combine into a tRNA with anti-codon GCC, where *G* at the first position of the anti-codon of the tRNA is paired with *U* at the third position of the triple code of an additional single strand $r_{t}^{\prime }2$. It implies that the wobble pairing rule G:U had been established as early as the end of the initiation stage of the roadmap. The transition from *C* to *T* occurred at the position #12, which resulted in the wobble pairing rule U:GorA. Taking y10r10 as a template, $y_{t}10$ with CCG is formed by the triplex base pairing, and $r_{t}10$ with CGG and $r_{t}^{\prime }10$ with UGG are also formed, where the transition from *C* to *U* occurred in the formation of $r_{t}^{\prime }10$. The complementary strands $y_{t}10$ and $r_{t}^{\prime }10$ combine into a tRNA with anti-codon UGG, where *U* at the first position of the anti-codon of the tRNA is paired with *G* at the third position of the triple code of an additional single strand $y_{t}10$. The above explanation of the wobble pairing rules by tRNA mutations is supported by the observations of nonsense suppressor. For instance, the wobble pairing rule C:A for a UGA suppressor can be established by a transition from *G* to *A* at the 24th position of $tRNA^{Trp}$. The wobble pairing rules G:UorC and U:GorA had been established early in the evolution of the genetic code, which continued to flourish so as to make full use of the short supply tRNAs.

The evolutionary relationship between tRNAs that corresponds to pairs of different amino acids can also be explained according to the evolution of tRNAs along the roadmap. For example, based on the substitution *G* to *A*, t16(AUG,Met) can evolve to t15(AUA,Ile), and based on the substitution *G* to *C*, t3(GAG,Glu) can evolve to t4(GAC,GAU,Asp), and so on (Figure 5c). However, this kind of evolution of tRNAs involves not only anti-codons but also para-codons because it inevitably needs extra help from aaRSs. There is a close relationship between the evolution of tRNAs and the biosynthetic families of amino acids, so the sequences of tRNAs coevolved with the sequences of aaRSs at each step of the roadmap. The recognition between tRNAs and aaRSs will be explained next, where there are many technical details, and each step needs to be straightened out in order to draw a comprehensive conclusion.

The evolution of tRNAs played significant roles to implement the number of canonical amino acids as 20. There is an important difference between the early prime tRNAs tn and the late derivative tRNAs $tn^{+}$. Generally speaking, the wobble pairing rules apply to the late derivative tRNAs $tn^{+}$ rather than to the early prime tRNAs tn (Figure 6b). The early prime tRNAs do not need wobble pairings so as to accurately implement the number of bases in codons as 3, whereas the late derivative tRNAs need wobble pairings so as to improve translation efficiency via codon degeneracy. This was a dynamic process to achieve that the number of canonical amino acids equals to the combination number of bases, which can hardly be fulfilled in lack of tRNAs but can be adjusted by choosing among the numerous candidates of tRNAs.

#### 2.3.3. Palindrome

Palindromic sequences play significant roles not only in contemporary molecular biology but also in the prebiotic evolution. Palindromic or non-palindromic codons on the roadmap can produce different effects in the origin and evolution of informative macromolecules. The cloverleaf secondary structure of tRNAs can be explained by the complementary palindrome in assembling tRNAs. Furthermore, the evolution of aaRSs also depended strongly on the evolution of palindromic para-codons along the roadmap, which will be explained next.

**Figure 8:**
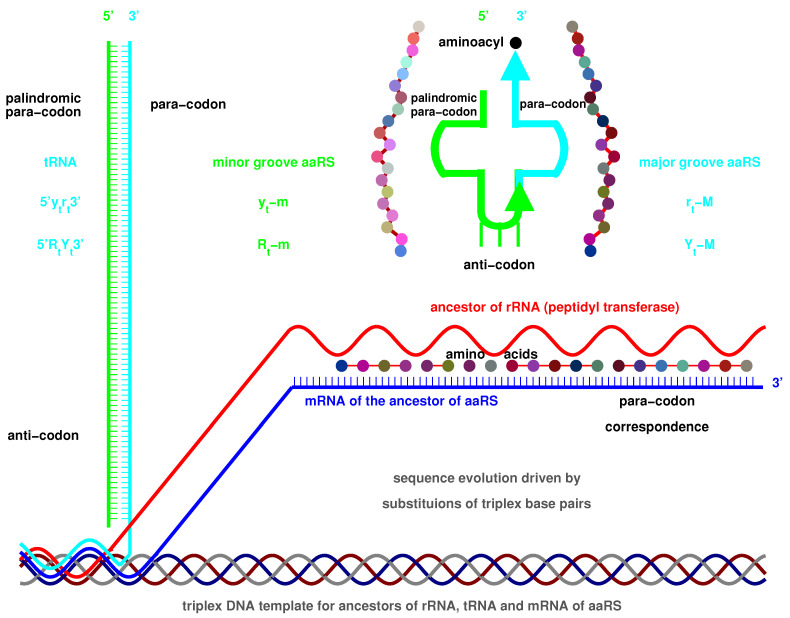
The origin and evolution of four classes of early aaRSs in the junior stage of the primordial translation mechanism in absent of tRNA and ribosome. The first aaRS can be produced through the non-random evolution of the triplex DNA and the corresponding RNAs. At the beginning of the translation mechanism, DNAs are the carrier of information, and RNAs develop the functions of life.

There are two types of tRNAs: type $5^{\prime }y_{t}r_{t}3^{\prime }$ and type $5^{\prime }R_{t}Y_{t}3^{\prime }$, where the two single RNA strands $y_{t}$ and $r_{t}$, $Y_{t}$ and $R_{t}$ are complementary to each other. A D-loop and an anti-codon loop situate in the $5^{\prime }$-end RNA strand ($y_{t}$ for type $5^{\prime }y_{t}r_{t}3^{\prime }$ and $R_{t}$ for type $5^{\prime }R_{t}Y_{t}3^{\prime }$), while a TΨC loop and a missing loop situate in the $3^{\prime }$-end RNA strand ($r_{t}$ for type $5^{\prime }y_{t}r_{t}3^{\prime }$ or $Y_{t}$ for type $5^{\prime }R_{t}Y_{t}3^{\prime }$) (Figure 6a). The strand pair $y_{t}$ and $r_{t}$ or $Y_{t}$ and $R_{t}$ can form two pairs of hairpins in the complementary double-stranded RNA, where the D-loop and the TΨC loop constitute a pair of hairpins, and the anti-codon loop and the missing complementary loop constitute another pair of hairpins (Figure 6a). When the missing loop has been deleted, the three other loops form a cloverleaf-shaped tRNA (Figure 6a). A palindromic nucleotide sequence can form a hairpin, and palindromic complementary double RNA sequences can form a pair of hairpins, which can account for the cloverleaf secondary structure of tRNAs (Figure 6a and Figure 8). If there are palindromic sequence intervals in the $5^{\prime }$-end RNA strand, there will also be the corresponding palindromic sequence intervals in the complementary $3^{\prime }$-end RNA strand. A D-loop and an anti-codon loop can form in the $5^{\prime }$-end RNA strand, owing to the complementarity in the palindromic sequence intervals. Accordingly, a TΨC loop and a missing loop can also form in the $3^{\prime }$-end RNA strand, which correspond to the D-loop and the anti-codon loop, respectively. After deleting the missing loop, a catenated RNA strand with three loops can form a cloverleaf secondary structure, and consequently, a stable tertiary structure can form. Therefore, palindromic sequences contribute to the formation of stable RNA structures in the prebiotic evolution. It is easy to generate palindromic oligonucleotides according to the base substitutions along the roadmap (Figure 5a,b). So, it tended to generate pairs of palindromic single RNA strands so as to assemble cloverleaf-shaped tRNA candidates. Numerous tRNA candidates can be produced by such an assembly line during the prebiotic evolution, where several qualified tRNAs with proper anti-codons and para-codons can be selected to carry the respective amino acids. Although it is difficult for the origin of aaRSs in the prebiotic evolution (Figure 8), it is not too difficult for the origin of tRNAs and amino acids. The early aaRSs had chance to adapt by choosing among the numerous tRNA candidates and amino acid candidates. Thus, the degree of difficulty for the origin of life can be reduced to some extent. Yet, if both tRNAs and aaRSs had been rare, there would have been little opportunity to establish the correspondence relationship between aaRSs and tRNAs.

### 2.4. Origin of aaRS

#### 2.4.1. Para-Codon

On one hand, an aaRS is able to recognise cognate tRNAs by para-codons (Figure 6b and Figure 8). On the other hand, the aaRS is able to catalyse the esterification of proper amino acid to its cognate tRNA (Figure 8). The origin of aaRS is one of the most difficult events in the origin of life because a primordial mechanism must be invented to generate the earliest proteins in absence of ribosome, and, meanwhile, aaRSs have to possess both para-codons and enzyme activity. It should be a rare critical event for the emergence of the first aaRS with enzyme activity in primordial sequence evolution. Following this process, the enzyme activity can transmit from the common ancestor of aaRSs to all the descendant aaRSs, either to the class I or class II aaRSs. Thus, the evolution of para-codons became to play a leading role in the evolution of aaRSs. The evolution of aaRS closely related to both the evolution of tRNA and the biosynthesis families of amino acids. The evolution of para-codons can be explained in the triplex picture. The para-codons of aaRSs coevolved with the sequences of tRNAs along the roadmap. The abilities to recognise certain amino acids came from the coevolution within the biosynthetic families of amino acids. According to the sequence evolution in the triplex picture, the recognition of tRNA by aaRS can be explained by the sequence homology between the template RNA of aaRS and the corresponding major or minor groove side sequence of tRNA. The recognition between aaRS and its template RNA led to the recognition between aaRS and the corresponding tRNA.

There are two types of tRNA according to the generation process of tRNA along the roadmap: type $5^{\prime }y_{t}r_{t}3^{\prime }$ and type $5^{\prime }R_{t}Y_{t}3^{\prime }$ (Figure 5a,b), where the $5^{\prime }$ side corresponds to the minor groove, while the $3^{\prime }$ side to the major groove. Additionally, the aaRSs can combine with the two types of tRNAs from either minor groove or major groove (Figure 5c and Figure 8). Thus, there are four classes of aaRSs: class $y_{t}-m$ aaRS, class $r_{t}-M$ aaRS, class $R_{t}-m$ aaRS, class $Y_{t}-M$ aaRS (Figure 5c and Figure 7). The four symbols indicate that aaRSs combine with tRNAs, respectively, from the minor groove (*m*) side $5^{\prime }y_{t}$ (*y*) of type $5^{\prime }y_{t}r_{t}3^{\prime }$ tRNA, from the major groove (*M*) side $r_{t}3^{\prime }$ (*r*) of type $5^{\prime }y_{t}r_{t}3^{\prime }$ tRNA, from the minor groove (*m*) side $5^{\prime }R_{t}$ (*R*) of type $5^{\prime }R_{t}Y_{t}3^{\prime }$ tRNA, and from the major groove (*M*) side $Y_{t}3^{\prime }$ (*Y*) of type $5^{\prime }R_{t}Y_{t}3^{\prime }$ tRNA.

The evolution of aaRSs occurred between the four classes of aaRSs (Figure 7). The sequences of para-codon can evolved between the homologous strands, and it can also evolve between the complementary strands when the sequences of para-codons are palindromic (Figure 7). According to the evolution of palindromic para-codons and the origin of the template RNA of aaRS (Figure 8), the class $y_{t}-m$ aaRS can be complementary with the class $r_{t}-M$ aaRS owing to the complementary two strands $5^{\prime }y_{t}$ and $r_{t}3^{\prime }$ that combine into the type $5^{\prime }y_{t}r_{t}3^{\prime }$ tRNA (Figure 5a), and the class $R_{t}-m$ aaRS can be complementary with the class $Y_{t}-M$ aaRS owing to the complementary two strands $5^{\prime }R_{t}$ and $Y_{t}3^{\prime }$ that combine into the type $5^{\prime }R_{t}Y_{t}3^{\prime }$ tRNA (Figure 5b). According to the evolution of palindromic para-codons and the coevolution of the template RNAs of aaRSs with tRNAs (Figure 7 and Figure 8), the class $r_{t}-M$ aaRS can be complementary with the class $Y_{t}-M$ aaRS, and the class $R_{t}-m$ aaRS can be complementary with the class $y_{t}-m$ aaRS. The class $y_{t}-m$ aaRS can be homologous to the class $Y_{t}-M$ aaRS, and the class $r_{t}-M$ aaRS can be homologous to the class $R_{t}-m$ aaRS. These relationships are useful for studying the evolution of aaRS along the roadmap.

The aaRSs are denoted in evolutionary order as aaRS1 to aaRS20 instead of GlyRS to LysRS for convenience, according to the recruitment order of the corresponding amino acids from No.1Gly to No.20Lys, respectively. The ancestor of aaRSs, namely the major groove aaRS1, belongs to the class $r_{t}-M$ aaRS, which catalysed pairing between the amino acid 1Gly and the tRNA t1 and which approaches to the type $5^{\prime }Y_{t}R_{t}3^{\prime }$ tRNA t1 from the major groove side $R_{t}3^{\prime }$ (Figure 7). The aaRS1 evolved into the same class aaRS2 and the $Y_{t}-M$ class aaRS7 (Figure 7). The aaRS2 evolved into aaRS3. According to the evolution of the Glu biosynthesis family, aaRS3 evolved into aaRS6, aaRS10, aaRS13, and, furthermore, aaRS14, and aaRS3 evolved into aaRS4 (Figure 7). According to the evolution of the Asp biosynthesis family, aaRS4 evolved into aaRS9, aaRS19, and, furthermore, aaRS15, aaRS16, and aaRS20 (Figure 7). According to the evolution of the Ser biosynthesis family, aaRS7 evolved into aaRS11 and aaRS12. According to the evolution of the Val biosynthesis family, aaRS2 evolved into aaRS5, aaRS8. According to the evolution of the Phe biosynthesis family, aaRS8 evolved into aaRS17 and aaRS18. In general, the evolutions via the Glu and Ser biosynthesis families took place in Hierarchy1 and Hierarchy2, corresponding to the codons whose second bases are *G* or *C*, while the evolutions via the Asp, Val and Phe biosynthesis families took place in Hierarchy3 and Hierarchy4, corresponding to the codons whose second bases are *A* or *U* (Figure 5c). This result accounts for the observation that the second bases of codons relate to the biosynthesis families of amino acids (Figure 4c).

The evolution of aaRSs depends strongly on the para-codon evolution (Figure 7 and Figure 8). Some para-codons of aaRS are homologous but not complementary to the previous para-codons. However, the para-codons of aaRSs that are complementary to the previous para-codons had to be palindromic. Some evolutions occurred between the same classes, which includes from aaRS1 to aaRS2, from aaRS3 to aaRS10, from aaRS15 to aaRS16, from aaRS4 to aaRS9, from aaRS4 to aaRS19, from aaRS8 to aaRS17 (Figure 7). Some evolutions of palindromic para-codons occurred between class $y_{t}-m$ and class $r_{t}-M$, which includes from aaRS2 to aaRS3, from aaRS2 to aaRS5, from aaRS3 to aaRS4, from aaRS9 to aaRS15, from aaRS19 to aaRS20 (Figure 7). Some evolutions of palindromic para-codons occurred between class $R_{t}-m$ and class $Y_{t}-M$, which includes from aaRS7 to aaRS11, from aaRS17 to aaRS18 (Figure 7). In addition, from aaRS1 to aaRS7 occurred between class $r_{t}-M$ and class $Y_{t}-M$; from aaRS2 to aaRS8 occurred between class $r_{t}-m$ and class $R_{t}-m$; from aaRS3 to aaRS6, from aaRS13 and from aaRS13 to aaRS14 occurred between class $y_{t}-m$ and class $Y_{t}-M$; from aaRS11 to aaRS12 occurred between class $R_{t}-m$ and class $y_{t}-m$ (Figure 7).

The evolution of aaRSs along the roadmap helps to clarify the traditional classifications of aaRSs in the literature (Figure 4c), such as the major groove (*M*), minor groove (*m*) classification [31], or the class *I* (IA, IB, IC), class II (IIA, IIB, IIC) classification (Gesteland et al. 2006). The four classes $y_{t}-m$, $r_{t}-M$, $R_{t}-m$, $Y_{t}-M$ classification here makes clear some confused ideas in the above classifications. The majority of class $r_{t}-M$ aaRSs correspond to class IIA aaRSs, and the majority of class $R_{t}-m$ aaRSs correspond to class IA aaRSs, which indicates an evolution from IIA to IA due to the reverse sequence relationship between the RNA templates of class $r_{t}-M$ aaRS and class $R_{t}-m$ aaRS (Figure 7). The majority of $Y_{t}-M$ aaRSs correspond to class IIA aaRSs, which were from the homologous $r_{t}-M$ aaRSs. In addition, the majority of class $y_{t}-m$ aaRSs correspond to class IA or IB aaRSs, which were from the complementary $r_{t}-M$ aaRSs due to evolution of palindromic para-codons (Figure 7). The traditional classification of aaRSs by the major groove and minor groove are reasonable in practice because the template RNAs of aaRSs are complementary between the major groove class and the minor groove class, where the para-codons are palindromic to link the two classes. Meanwhile, the traditional classification of aaRS by classes *A*, *B*, and *C* reflects some reasonable evolutionary relationships between aaRSs based on the evolution of the biosynthetic families.

#### 2.4.2. Coevolution of tRNA with aaRS

A comprehensive study of the evolution of the genetic code inevitably involves the origins of tRNAs and aaRSs. The intricate evolutionary relationships between tRNAs and aaRSs can be explained step by step for each codon in the triplex picture (Figure 7). The initiation stage on the roadmap played a fundamental role. At the end of the initiation stage, arbitrary finite sequences can be generated, which provided opportunities to generate complex RNAs, such as tRNAs, the template RNAs for aaRSs, ribozymes and the prototype of rRNAs, coding and non-coding RNAs, etc. The primordial translation mechanism were invented during the evolution of the genetic code. There were a junior stage and a senior stage of the primordial translation mechanism (Figure 8). The ancestor of aaRSs originated in the junior stage when no tRNAs were involved (Figure 8). However, the tRNAs and ribosomes were indispensable in the senior stage of the primordial translation mechanism, as well as in the modern translation mechanism. Certainly, the translation efficiency was low in the junior stage, was medium in the senior stage, and was high in the modern translation mechanism. There exists non-standard translation in experiments, such as direct translation from DNA to protein [60,61].

The benefits to explain the origins of tRNAs and aaRSs in the triplex picture are as follows. First, the ancestors of tRNAs and aaRSs did not originate from the random sequences; the sequence evolution along the roadmap was recurrent so the informative molecules were generated recurrently and accumulated in the prebiotic surroundings. Second, the evolutionary relationships between tRNAs and aaRSs can be naturally explained by the relationships of the homologous strands of the evolving triplex DNAs. The sequence of the template of the ancestor aaRS can be generated in the triplex picture by the junior stage of the primordial translation mechanism; meanwhile, the sequence of ribozyme can also be generated by the other strand of the same triplex nucleic acid. Thus, the earliest proteins, such as the ancestor of aaRSs, can be generated by the complex consisting of the ribozyme, the RNA template of aaRS, as well as a triplex DNA. Such a complex itself was the product of sequence evolution of triplex nucleic acids based on specific substitutions of triplex base pairs, where both the sequence for ribozyme and the sequence for the template of ancestor aaRS with enzyme activity were generated in different strands of the same triplex DNA by chance. Although the efficiency to produce proteins was low in this junior stage, it was feasible to generate a small number of proteins by this complex consisting only nucleic acids. The ancestor of aaRS with enzyme activity can be generated by this complex, which naturally tends to combine with the corresponding RNA template.

If the sequence of tRNA is homologous to the above RNA template, the ancestor aaRS also tends to combine with the tRNA. Furthermore, the above requirement can be reduced to homologous para-codons. Thus, in the triplex picture, the aaRSs coevolved with the para-codons, while the tRNAs coevolved with the codons. When considering the homologous or complementary sequence relationships, the reverse sequence relationships and the base substitution relationships in the strands of triplex nucleic acids, the intricate evolutionary relationships between tRNAs and aaRSs can be revealed in detail (Figure 5c and Figure 7). It is more difficult to generate aaRSs than to generate tRNAs, so there existed numerous tRNAs candidates in the prebiotic surroundings. Only the tRNAs that were recognised by aaRSs can be recruited into the living system. For example, the RNA $5^{\prime }-y_{t}1r_{t}1-3^{\prime }$ were recognised by the class $r_{t}-M$aaRS1, so it was chosen as the first tRNA t1 to transport 1Gly. The prime RNAs tn were recognised by aaRSn, so they were chosen as the tRNAs to transport No.n amino acids (Figure 5c and Figure 7), respectively. Similarly, the derivative RNAs $tn{}^{\prime }$, $tn^{+}$, $tn^{+}{}^{\prime }$, $tn^{-}$, $tn^{-}{}^{\prime }$, with non-palindromic or palindromic para-codons homologous to the para-codons of tn, were recognised by aaRSn, so they became the tRNAs to transport No.n amino acids, respectively. Para-codons are the key factors for the recognition between tRNAs and aaRSs. The types of tRNAs are not necessarily same for the cognate tRNAs. Generally, the aaRSs combine with the cognate tRNAs from the same side. For example, aaRS8 combines with the $5^{\prime }R_{t}Y_{t}3^{\prime }$ type cognate tRNAs t8, $t8{}^{\prime }$, $t8^{+}$, $t8^{-}$, and $t8^{-}{}^{\prime }$ from the minor groove side, where the para-codons can be non-palindromic (Figure 7); aaRS7 combines with the $5^{\prime }R_{t}Y_{t}3^{\prime }$ type tRNAs t7, $t7^{+}$, $t7^{-}$ and the $5^{\prime }y_{t}r_{t}3^{\prime }$ type tRNAs $t7^{-}{}^{\prime }$ from the major groove side, where the para-codons of the two types of tRNAs have to be palindromic (Figure 7). However, aaRS10 combines with the $5^{\prime }y_{t}r_{t}3^{\prime }$ type tRNAs t10, $t10{}^{\prime }$ and the $5^{\prime }R_{t}Y_{t}3^{\prime }$ type tRNA $t10^{+}$ from the minor groove side, while combine with the $5^{\prime }y_{t}r_{t}3^{\prime }$ type tRNAs $t10^{-}$ and $t10^{-}{}^{\prime }$ from the major groove side, where the para-codons also need to be palindromic (Figure 7).

The biosynthetic families played essential roles in the evolution of aaRSs when both anti-codon and para-codon had changed (Figure 7). There were far more than 20 amino acids in the prebiotic surroundings. Only the amino acids that were recognised by aaRSs can be recruited into the living system. When aaRS1 involved to aaRS2, aaRS2 recognised 2Ala, as well as t2, from the major groove side, which inherited from aaRS1 that recognised 1Gly, as well as t1, from the major groove side. When aaRS2 involved to aaRS3, aaRS3 recognised 3Glu, as well as t3, from the minor groove side owing to the palindromic para-codons, which inherited from aaRS2 that recognised 2Ala, as well as t2, from the major groove side. When aaRSs involved in the same biosynthetic families: Glu family, Asp family, Val family, Ser family, and Phe family, the new aaRSs tended to recruit the new amino acids with the similar chemical properties in the same biosynthetic family. When aaRSs evolved from aaRS1 to aaRS20, the enzyme activity transmitted between the aaRSs, and the recognised tRNAs t1 to t20 and the recognised amino acids No.1Gly to No.20Lys were recruited, where the evolving non-palindromic or palindromic para-codons linked these evolutions.

The evolutionary pairs of aaRSs combining two sides of the same tRNAs along the roadmap agree with the results based on structures: IleRS and ThrRS, GlnRS (GluRS) and AspRS, and TyrRS and PheRS [4,62], and additionally SerRS and CysRS. The aaRS pair ThrRS and IleRS (namely aaRS9 and aaRS15) corresponds to an evolution from $r_{t}-M$aaRS9 to $y_{t}-m$aaRS15. The aaRS pair GluRS and AspRS (namely aaRS3 and aaRS4) corresponds to an evolution from $y_{t}-m$aaRS3 to $r_{t}-M$aaRS4. The aaRS pair PheRS and TyrRS (namely aaRS17 and aaRS18) corresponds to an evolution from $R_{t}-m$aaRS17 to $Y_{t}-M$aaRS18. The aaRS pair SerRS and CysRS (namely aaRS7 and aaRS11) corresponds to an evolution from $Y_{t}-M$aaRS7 to $R_{t}-m$aaRS11.

**Figure 9:**
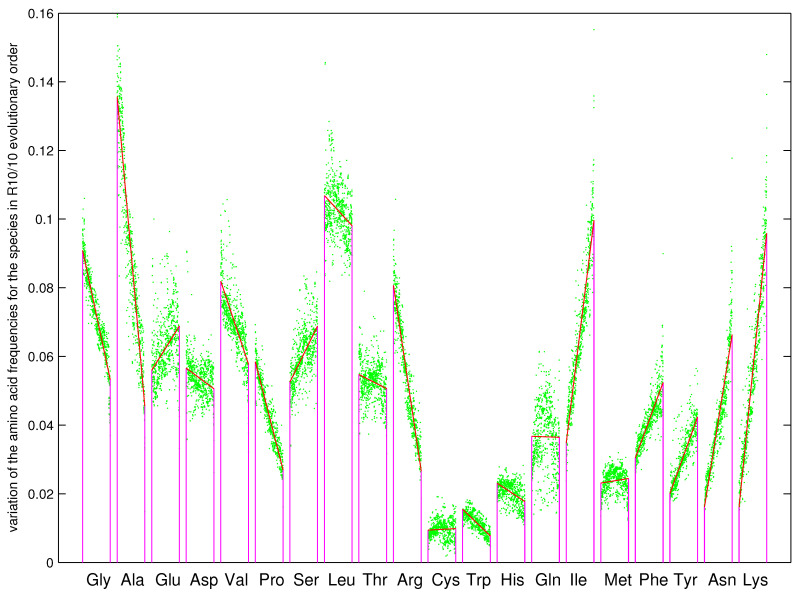
The recruitment orders of amino acids and codon pairs on the roadmap are supported by the variation of the amino acid frequencies. The 20 amino acids are arranged in the recruitment order on the roadmap Figure 1a. The 20 amino acid frequencies for each of the 803 species are obtained, respectively, based on the genomic data in NCBI. The 803 amino acid frequencies (green dots) for each of the 20 amino acids are all arranged properly in the $R_{10/10}$ order [36], respectively. The variation trend of the amino acid frequencies for each of the 20 amino acids is obtained by the regression line (denoted in red). Generally speaking, the variation trends for the earlier amino acids tend to decrease, and the variation trends for the latecomers to increase.

The recruitment order of the 20 amino acids from No.1 to No.20 can be obtained by the roadmap (Figure 3a and Figure 9), which meets the basic requirement that Phase I amino acids appeared earlier than the Phase II amino acids [1,2]. The species with complete genome sequences are sorted by the order $R_{10/10}$ according to their amino acid frequencies, where the order $R_{10/10}$ is defined as the ratio of the average amino acid frequencies for the last 10 amino acids to that for the first 10 amino acids [8,36,63,64,65]. Along the evolutionary direction indicated by the increasing $R_{10/10}$, the amino acid frequencies vary in different monotonous manners for the 20 amino acids, respectively (Figure 9). For the early amino acids Gly, Ala, Asp, Val, Pro, the amino acid frequencies tend to decrease greatly, except for Glu to increase slightly (Figure 9); for the midterm amino acids Ser, Leu, Thr, Cys, Trp, His, Gln, the amino acid frequencies tend to vary slightly, except for Arg to decrease greatly (Figure 9); for the late amino acids Ile, Phe, Tyr, Asn, Lys, the amino acid frequencies tend to increase greatly, except for Met to increase slightly (Figure 9). In the recruitment order from No.1 to No.20, the variation trends of the amino acid frequencies increase in general; namely, the later the amino acids recruited, the more greatly the amino acid frequencies tend to increase (Figure 9). The recruitment order of the amino acids from No.1 to No.20 is supported not only by the previous roadmap theory but also by this pattern of amino acid frequencies based on genomic data.

### 2.5. Recruitment of Codons

The roadmap only provided a logical substitution relationship of the 64 codons based on the stabilities of triplex base pairs (Figure 1a). It was the tRNAs and aaRSs that gave the genetic significance to the 64 codons (Figure 5c). The pair connections and route dualities observed in the recruitment of codons along the roadmap should be explained based on the coevolution of tRNAs with aaRSs (Figure 5b and Figure 7). The standard genetic code table can be comprehended in a biological context. Incidentally, the non-standard codons can also be explained.

#### 2.5.1. Pair Connection

The pair connections can be explained by the coevolution of tRNAs with aaRSs when aaRSn recognise, respectively, both the prime tRNAs tn (in bold in the following pair connections and route dualities) and the corresponding derivative tRNAs $tn^{\prime }$, $tn^{+}{}^{\prime }$ and $tn^{-}{}^{\prime }$, where the anti-codons of tRNAs change but the para-codons of tRNAs do not change, or when tn have the efficient ability to recognise similar codons by wobble pairings (Figure 7c and Figure 7). Taking #1−1Gly−#3 as an example, the $5^{\prime }y_{t}r_{t}3^{\prime }$ type tRNA t1 and the class $r_{t}-M$aaRS1 originated at #1 on the roadmap, and the same type tRNA $t1^{\prime }$ appeared at #3 on the roadmap. The aaRS1 for 1Gly can recognise both the same type tRNAs t1 and $t1^{\prime }$ via the same para-codon. Namely, tRNAs t1 and $t1^{\prime }$ recognise, respectively, the codons GGG at #1 and GGA at #3 on the purine stands (*R*) on the roadmap (Figure 5c).

The following pair connections are due to wobble pairings or the tRNA evolution from tn to $tn^{\prime }$, both of which can be recognised by the respective same aaRSn (Figure 5c, Figure 6b and Figure 7).
1Gly, aaRS1, **t1**→t1’: **#1 R**-Gly-#3 R2Ala, aaRS2, **t2**→t2’: **#7 R**-Ala-#9 R3Glu, aaRS3, **t3**→t3’: **#4 R**-Glu-#23 R4Asp, aaRS4, **t4** wobbling: **#5 R**-Asp-#21 R5Val, aaRS5, **t5**→t5’: **#19 R**-Val-#24 R6Pro, aaRS6, **t6** wobbling: **#1 Y**-Pro-#11 Y7Ser, aaRS7, **t7** wobbling: **#3 Y**-Ser-#14 Y8Leu, aaRS8, **t8**→t8’: **#20 Y**-Leu-#25 Y9Thr, aaRS9, **t9**→t9’: **#16 R**-Thr-#18 R10Arg, aaRS10, **t10**→t10’: **#10 R**-Arg-#13 R11Cys, aaRS11, **t11** wobbling: **#9 Y**-Cys-#18 Y12Trp, aaRS12, **t12** wobbling: **#12 R**-Trp-#(15 R)13His, aaRS13, **t13** wobbling: **#19 Y**-His-**#22 Y**14Gln, aaRS14, **t14**→t14’: **#20 R**-Gln-#28 R15Ile/16Met,aaRS15/16,**t15**/**t16**:**#29R**-Ile/Met-**#22R**17Phe, aaRS17, **t17** wobbling: **#23 Y**-Phe-#32 Y18Tyr, aaRS18, **t18** wobbling: **#24 Y**-Tyr-#29 Y19Asn, aaRS19, **t19** wobbling: **#26 R**-Asn-#30 R20Lys, aaRS20, **t20**→t20’: **#27 R**-Lys-#32 Rstop, no aaRS, no tRNA: **#25 R**-stop-#31 R


Especially, in the pair connection #29R−Ile/Met−#22R, aaRS15 for 15Ile evolved to aaRS16 for 16Met, and the corresponding t15 evolved to t16 by changing both anti-codon and para-codon.

The following pair connections are due to wobble pairings or the tRNA evolution from $tn^{+}$ to $tn^{+}{}^{\prime }$, both of which can be recognised by the respective same aaRSn (Figure 5c, Figure 6b, and Figure 7).
1Gly, aaRS1, $t1^{+}$ wobbling: #2 R-Gly-#6 R2Ala, aaRS2, $t2^{+}$ wobbling: #2 Y-Ala-#8 Y5Val, aaRS5, $t5^{+}$ wobbling: #5 Y-Val-#26 Y6Pro, aaRS6, $t6^{+}$ →$t6^{+}{}^{\prime }$: #10 Y-Pro-#12 Y7Ser, aaRS7, $t7^{+}$ →$t7^{+}{}^{\prime }$: #13 Y-Ser-#15 Y8Leu, aaRS8, $t8^{+}$ wobbling: #4 Y-Leu-#27 Y9Thr, aaRS9, $t9^{+}$ wobbling: #6 Y-Thr-#17 Y10Arg, aaRS10, $t10^{+}$ wobbling: #7 Y-Arg-#16 Y15Ile, aaRS15, $t15^{+}$ wobbling: #21 Y-Ile-#30 Y


The following pair connections are due to wobble pairings or the tRNA evolution from $tn^{-}$ to $tn^{-}{}^{\prime }$, both of which can be recognised by the respective same aaRSn (Figure 5c, Figure 6b, and Figure 7).
7Ser, aaRS7, $t7^{-}$ wobbling: #8 R-Ser-#17 R8Leu, aaRS8, $t8^{-}$ → $t8^{-}{}^{\prime }$: #28 Y-Leu-#31 Y10Arg, aaRS10, $t10^{-}$ → $t10^{-}{}^{\prime }$: #11 R-Arg-#14 R


The pair connections between non-standard codons are also due to the non-standard tRNA evolution. The non-standard tRNAs tn∗ with non-standard anti-codons can also be recognised by aaRSn. The existence of non-standard codons indicates a variety of possibilities to choose tRNAs among the candidate tRNAs by the aaRSs during the evolution of the genetic code. The non-standard genetic code system can exist in case of certain metabolic cycle (Figure 5c and Figure 7).
7Ser, aaRS7, $t7^{*}$ → $t7^{*}{}^{\prime }$: #11 R-Ser-#14 Rstop, no aaRS, no tRNA: #11 R-Ser-#14 R9Thr, aaRS9, $t9^{*}$ wobbling: #4 Y-Thr-#27 Y9Thr, aaRS9, $t9^{*+}$ →$t9^{*+}{}^{\prime }$: #20 Y-Thr-#25 Y14Gln, aaRS14, $t14^{*}$ → $t14^{*}{}^{\prime }$: #25 R-Gln-#31 R


#### 2.5.2. Route Duality

Route duality refers to the relationships between pair connections in different routes. The route duality can also be explained by the coevolution of tRNAs with aaRSs when aaRSn recognise both the prime tRNAs tn and the corresponding derivative tRNAs $tn^{+}$ and $tn^{-}$, respectively. Taking the route duality #7−Ala−#9∼#2−Ala−#8, for example, there were two pair connections: #7−Ala−#9 connecting via the $5^{\prime }y_{t}r_{t}3^{\prime }$ type tRNA t2, $t2^{\prime }$ and #2−Ala−#8 connecting via the $5^{\prime }R_{t}Y_{t}3^{\prime }$ type tRNA $t2^{+}$. The route duality between #7−Ala−#9 in Route2 and #2−Ala−#8 in Route1 is due to the fact that aaRS2 for 2Ala recognises both the tRNAs t2, $t2^{\prime }$ and the different type tRNAs $t2^{+}$ by same para-codon.

The following route dualities are due to the tRNA evolution from tn to $tn^{+}$ or $tn^{-}$, all of which can be recognised by the respective same aaRSn (Figure 5c, Figure 6b and Figure 7).
1Gly, aaRS1, **t1** → $t1^{+}$**#1**-Gly-#3 (Route 0) ∼ #2-Gly-#6 (Route 1)2Ala, aaRS2, **t2** → $t2^{+}$**#7**-Ala-#9 (Route 2) ∼ #2-Ala-#8 (Route 1)5Val, aaRS5, **t5** → $t5^{+}$**#19**-Val-#24 (Route 2) ∼ #5-Val-#26 (Route 1)6Pro, aaRS6, **t6** → $t6^{+}$**#1**-Pro-#11 (Route 0) ∼ #10-Pro-#12 (Route 3)7Ser, aaRS7, **t7** → $t7^{+}$**#3**-Ser-#14 (Route 0) ∼ #13-Ser-#15 (Route 3)            and **t7** → $t7^{-}$**#3**-Ser-#14 (Route 0) ∼ #8-Ser-#17 (Route 1)8Leu, aaRS8, **t8** → $t8^{+}$**#20**-Leu-#25 (Route 3) ∼ #4-Leu-#27 (Route 0)            and **t8** → $t8^{-}$**#20**-Leu-#25 (Route 3) ∼ #28-Leu-#31 (Route 3)9Thr, aaRS9, **t9** → $t9^{+}$**#16**-Thr-#18 (Route 2) ∼ #6-Thr-#17 (Route 1)10Arg, aaRS10, **t10** → $t10^{+}$**#10**-Arg-#13 (Route 3) ∼ #7-Arg-#16 (Route 2)            and **t10** → $t10^{-}$**#10**-Arg-#13 (Route 3) ∼ #11-Arg-#14 (Route 0)

The relationship between pair connections via aaRS evolution can be regarded as quasi route dualities (Figure 5c, Figure 6b and Figure 7).
3Glu/4Asp, t3/t4, aaRS3 → aaRS4**#4**-Glu-#23 (Route 0) ∼**#5**-Asp-#21 (Route 1)7Ser/10Arg, $t7^{-}$/$t10^{-}$, aaRS7 / aaRS10#8-Ser-#17 (Route 1) ∼ #11-Arg-#14 (Route 0)11Cys/12Trp, t11/t12, aaRS11 → aaRS12**#9**-Cys-#18 (Route 2) ∼**#12**-Trp-(#15) (Route 3)13His/14Gln, t13/t14, aaRS13 → aaRS14**#19**-His-#22 (Route 2) ∼**#20**-Gln-#28 (Route 3)15Ile/16Met,t15,t16/$t15^{+}$,aaRS15→aaRS16**#29**-Ile/Met-**#22** (Route 2) ∼ #21-Ile-#30 (Route 1)8Leu/17Phe, $t8^{-}$/t17, aaRS8 → aaRS17#28-Leu-#31 (Route 3) ∼ **#23**-Phe-#32 (Route 0)18Tyr/stop, t18, aaRS18**#24**-Tyr-#29 (Route 2) ∼**#25**-stop-#31 (Route 3)19Asn/20Lys, t19/t20, aaRS19 → aaRS20**#26**-Asn-#30 (Route 1) ∼**#27**-Lys-#32 (Route 0)

The route dualities between non-standard pair connections are also due to the non-standard tRNA evolution. The non-standard tRNAs $tn^{*}$ and $tn^{*+}$ with non-standard anti-codons can also be recognised by the respective same aaRSn (Figure 5c and Figure 7). The phenomenon of non-standard genetic code is due to alternative choice of tRNAs by aaRSs as small probability events in the fulfilment of the genetic code.
7Ser, aaRS7, $t7^{-}$ → $t7^{*}$#8-Ser-#17 (Route 1) ∼ #11-(Ser)-#14 (Route 0)9Thr, aaRS9, $t9^{*}$ → $t9^{*+}$#4-(Thr)-#27 (Route 0) ∼ #20-(Thr)-#25 (Route 3)stop#11-(stop)-#14 (Route 0) ∼ #15-stop-#31 (Route 3)

The 4×4 codon boxes in the standard genetic code table come from the 8 route dualities and the 8 quasi route dualities (Table 2 and Figure 4a,b), where the pair connections are from Hierarchy1 to Hierarchy2, from Hierarchy2 to Hierarchy3, and from Hierarchy3 to Hierarchy4, only. And the route dualities only exist between Route0 and Route1, between Route2 and Route3, between Route0 and Route3, and between Route1 and Route2, but not between Route0 and Route2 and Route1 and Route3 (Figure 4a,b).

### 2.6. Codon Degeneracy

The degeneracies 6, 4, 3, 2, or 1 for the 20 amino acids can be explained one by one according to pair connections and route dualities on the roadmap based on the coevolution of tRNAs with aaRSs in the triplex picture (Figure 5c, Figure 6b and Figure 7). Especially, the evolution of aaRSs based on the biosynthetic families played significant roles in the expansion of the genetic code. The degeneracy 2 mainly results from pair connections. The degeneracy 4 or 6 mainly result from the expansion of the genetic code from the initial subset by route dualities for Ser, Leu, Ala, Val, Pro, and Thr (Figure 3a,b).

The degeneracy 6 for Ser, Leu, and Arg can be explained by pair connections and route dualities (Figure 1a, Figure 3b, Figure 5c, Figure 6b and Figure 7), where Ser and Leu belong to the initial subset, and Arg was recruited immediately after the initial subset. All of them have appeared in Route0. The 6 codons of Ser satisfy both the route duality and pair connection

#3−Ser−#14∼#13−Ser−#15 and #8−Ser−#17.

The 6 codons of Leu satisfy both the route duality and pair connection

#20−Leu−#25∼#4−Leu−#27 and #28−Leu−#31.

The 6 codons of Arg satisfy both the route duality and pair connection

#10−Arg−#13∼#7−Arg−#16 and #11−Arg−#14.

The degeneracy 4 for Gly, Ala, Val, Pro, and Thr can be explained by route dualities (Figure 1a and Figure 3b). All of them belong to the initial subset. The degeneracy 4 for Gly satisfy the route duality:

#1−Gly−#3∼#2−Gly−#6.

The degeneracy 4 for Ala satisfy the route duality:

#2−Ala−#8∼#7−Ala−#9.

The degeneracy 4 for Val satisfy the route duality:

#5−Val−#26∼#19−Val−#24.

The degeneracy 4 for Pro satisfy the route duality:

#1−Pro−#11∼#10−Pro−#12.

The degeneracy 4 for Thr satisfy the route duality:

#6−Thr−#17∼#16−Thr−#18.

The degeneracy 2 for Glu, Asp, Cys, His, Gln, Phe, Tyr, Asn, and Lys can be explained by pair connections (Figure 1a and Figure 3b). They satisfy the following pair connections, respectively: #4−Glu−#23, #5−Asp−#21, #9−Cys−#18, #19−His−#22, #20−Gln−#28, #23−Phe−#32, #24−Tyr−#29, #26−Asn−#30, #27−Lys−#32. The degeneracy 3 for Ile and the degeneracy 1 for Met satisfies the route duality (Figure 1a, Figure 3b, Figure 5c, Figure 6b and Figure 7).

#21−Ile−#30∼#22−Met/Ile−#29.

The degeneracy 1 for Trp satisfies the pair connection for nonstandard genetic code #12−Trp/stop(Trp)−#15. This pair connection includes a stop codon; the other stop codons satisfy the pair connection: #25−stop−#31 (Figure 1a, Figure 3b, Figure 5c, Figure 6b and Figure 7).

## 3. Results

### 3.1. Driving Force in the Prebiotic Sequence Evolution

First, I propose an elegant roadmap for the evolution of the genetic code (Figure 1a). Around the middle of the last century, double helix DNAs, the genetic code, as well as triplex DNAs, were discovered, the former two of which greatly enhanced our understanding of life. There are indeed profound relationships among the above three discoveries. Although triple-helical nucleic acids are rare in vivo, they might be the unsung heroes in the origin of life. According to the substitutions of triplex base pairs from weak to strong along the roadmap, the recruitment of the 64 codons has been described from initiation to expansion and, finally, to the ending, and, hence, the perplexing codon degeneracy has been obtained.

The whole process is complicated and cumbersome, and has been explained step by step in the Methods section. Here is an overview of the basic process. Concretely speaking, the stability of the 16 triplex base pairs in triplex DNAs are from instability (−), weak (+) to strong (++, 3+, 4+) [10,11]. This stability order in experiments is crucial to establish a roadmap for the evolution of the genetic code. PolyC·PolyG∗PolyG is a common and easily formed YR∗R triplex DNA [10,13], which is bound together by triplex base pair CG∗G. The sequences evolved via substitutions between triplex base pairs when the strands of triplex DNAs combined and separated alternatively. Only three kinds of substitutions between triplex base pairs are practically required to obtain a complete set of 64 codons on the roadmap (Figure 1 and Figure 2): (1) substitution of (+)CG∗G by (++)CG∗A (transition from *G* to *A* with increasing stability from + to ++). This is the most common substitution on the roadmap by which all the codons in Route0 and most codons in Route1∼3 were recruited; (2) substitution of (+)CG∗G by (4+)CG∗C (transversion from *G* to *C* with increasing stability from + to 4+), which blazed a new path at #2, #7, #10 for the recruitment of codons in Route1∼3, respectively; (3) substitution of (+)GC∗C by (++)GC∗T (transition from *C* to *T* with increasing stability from + to ++) at #6, #19, #12, by which the remaining codons in Route1∼3 were recruited.

Hence, a roadmap has been obtained with 4 Routes and 4 Hierarchies (Figure 1a, Figure 3b and Figure 4a). This unique roadmap has narrowly avoided those unstable triplex base pairs that can hinder the sequence evolution of triplex DNAs. The roadmap describes recruitments of both the 64 codons and the 20 amino acids in proper order during coevolution of tRNAs with aaRSs. The initial codon pair GGG·CCC (#1) corresponds the amino acid pair Gly and Pro, and the consequent codon pair GGC·GCC (*G* to *C* at #2) corresponds a new amino acid pair Gly and Ala. The obtained pair connection #1−Gly−#2 indicates that the common Gly is encoded by GGG in the former pair and GGC in the latter pair. Pair connections appear step by step along the roadmap, which relates to the evolution of the corresponding tRNAs. In addition, there are route dualities between pair connections, which relate to the evolution of the corresponding aaRSs. The expansion of codons along the roadmap has been explained by route dualities from the Phase I amino acids [34] Ala, Val, Pro, Ser, Leu, and Thr, which are due to recognition of tRNAs by the corresponding aaRSs step by step. In addition, stop codons and non-standard genetic code often occur at the ending stage. Thus, the intricate codon degeneracy has been obtained based on the incremental stability of triplex base pairs. In the triplex picture for the prebiotic evolution, the base substitution of triplex DNA drives both the recruitment of the 64 codons and the corresponding coevolution of tRNAs and aaRSs, step by step.

The benefit of the triplex picture is that nonrandom sequences can be generated routinely in the prebiotic evolution. The modification of homopolymers became a routine process in forming the codon degeneracy. This non-living apparatus based on sequence evolution of triplex DNAs was able to maintain during geologically long period, by which similar nonrandom sequences can be statistically generated again and again under selective pressure at any appropriate time. Hence, the nonrandom sequences, e.g., tRNAs and aaRSs, were able to emerge more efficiently than any mechanism to choose informative molecules from random sequences. Such an HfC-like apparatus based on sequence evolution of triplex DNAs had vanished after the establishment of the genetic code system, whose relic may have remained in the triplex base pairs in tRNAs at present.

### 3.2. Explanation of Two Classes of aaRSs According to Coevolution of tRNAs with aaRSs

Then, I explain the coevolution of tRNAs with aaRSs (Figure 5, Figure 6 andFigure 7), by which the two classes of aaRSs [31] and the anti-codons and para-codons of tRNAs have been explained in detail. A comprehensive study of the evolution of the genetic code inevitably involves the intricate evolutionary relationships between tRNAs and aaRSs. The evolution of triple-helical nucleic acids D·D∗D and D·D∗R (*D* for DNA, *R* for RNA) [10] created conditions for coevolution of tRNAs and aaRSs along the roadmap. The third RNA strand *R* and its complementary strand can carry codons and anti-codons in sequence evolution along the roadmap, which, hence, accounts for that the tRNAs can be assembled by pairs of these complementary RNAs [66] whose anti-codons evolved along the roadmap (Figure 5a,b and Figure 6a). Meanwhile, genes of aaRSs also evolved along the roadmap, which were homologous to the complementary [67,68] templates of major or minor groove sides of tRNAs. The recognition of a tRNA by certain aaRS came from the combining ability between the aaRS and its gene that is homologous to the corresponding side of the tRNA. Hence, the recognition of tRNAs by aaRSs kept pace with the evolution of the genetic code along the roadmap. The tRNAs were relatively easy to be assembled, so there existed numerous candidate tRNAs. Only tRNAs that were recognised by aaRSs had been recruited into the living system. The genes of aaRSs are scarce, whose enzyme activity came from a common ancestor. The genes of the two classes of aaRSs evolved alternatively in two complementary strands. Palindrome enabled recognition of tRNA via choosing its appropriate side by the corresponding aaRS.

The intricate relationships between tRNAs and aaRSs along the roadmap has been explained, which agrees with both the anti-codons of tRNAs and the two classes of aaRSs in observations (Figure 5). The evolution of aaRSs along the roadmap in the triplex picture helps to clarify the traditional classifications of aaRSs in the literature. The major/minor groove classification of aaRSs [31] can be accounted for by the complementary strands of the template RNAs of aaRSs, and the *A*/*B*/*C* sub-classification of aaRSs [69] relates to the impact from biosynthetic families of amino acids. In most cases, the aaRSs combine with the cognate tRNAs from the same side, whose classes are fixed. As a special case, aaRS10(ArgRS) combines with the $5^{\prime }y_{t}r_{t}3^{\prime }$ type tRNAs t10, $t10{}^{\prime }$ and the $5^{\prime }R_{t}Y_{t}3^{\prime }$ type tRNA $t10^{+}$ from the minor groove side, while combine with the $5^{\prime }y_{t}r_{t}3^{\prime }$ type tRNAs $t10^{-}$ and $t10^{-}{}^{\prime }$ from the major groove side, where the para-codons need to be palindromic. In the evolution from aaRS1(GlyRS) to aaRS2(AlaRS), for instance, aaRS2(AlaRS) recognised 2Ala from major groove side of t2, whose class follows the former aaRS1(GlyRS) to recognise 1Gly from major groove side of t1. In addition, in the consequent evolution from aaRS2(AlaRS) to aaRS3(GluRS), aaRS3(GluRS) recognised 3Glu yet from minor groove side of t3 due to the palindromic para-codons. The biosynthetic families played significant roles in the evolution of aaRSs when both anti-codon and palindromic or non-palindromic para-codon evolved. When aaRSs involved in the same biosynthetic families, the new aaRSs tended to recruit amino acids in same biosynthetic family with similar chemical properties. Thus, the observed recognition of tRNAs from major or minor groove sides by aaRSs have been explained for respective amino acids in detail (Figure 7). The aaRS pair supposed to combine both sides of tRNA simultaneously [4,62] should be amended as new aaRS pair that combined one side of a tRNA and evolved to the other side. The pairs IleRS-ThrRS and TyrRS-PheRS appear both in the above literature and here. However, the pair GluRS-ThrRS in the above literature should be changed to GlnRS-ThrRS. In addition, the pair SerRS-CysRS appeared here was missing in the above literature.

### 3.3. Explanation of the Codon Degeneracy on the Genetic Code Chart

As the main result, the codon degeneracy should be explained based on the roadmap for the evolution of the genetic code (Figure 1) and the coevolution of tRNAs with aaRSs (Figure 5 and Figure 7). The intricate codon degeneracies are just the relics of learning process for the recognition of tRNAs by aaRSs. The pair connections and route dualities on the roadmap result from the evolution of tRNAs and the recognition of tRNAs by aaRSs (Figure 5). Especially, homologous aaRSs often evolved within the biosynthetic families of amino acids by combining either the same side or the opposite side of tRNAs (Figure 7). The 4×4 codon boxes in the standard genetic code table came from the 8 route dualities and the 8 quasi route dualities (Figure 1).

The degeneracies 6, 4, 3, 2, or 1 for the 20 amino acids have been explained, respectively, according to the corresponding pair connections and route dualities (Figure 1, Figure 5 and Figure 7). The large degeneracy 4 or 6 mainly results from the expansion of codons for the amino acids recruited in the initiation stage: Ser, Leu, Ala, Val, Pro, and Thr. The degeneracy 6 for Ser, Leu, and Arg is due to the following route dualities and pair connections, respectively: #3−Ser−#14∼#13−Ser−#15 and #8−Ser−#17, #20−Leu−#25∼#4−Leu−#27 and #28−Leu−#31, #10−Arg−#13∼#7−Arg−#16 and #11−Arg−#14. The degeneracy 4 for Gly, Ala, Val, Pro and Thr is due to the following route dualities, respectively: #1−Gly−#3∼#2−Gly−#6, #2−Ala−#8∼#7−Ala−#9, #5−Val−#26∼#19−Val−#24, #1−Pro−#11∼#10−Pro−#12, #6−Thr−#17∼#16−Thr−#18. In addition, the degeneracy 2 for Glu, Asp, Cys, His, Gln, Phe, Tyr, Asn, and Lys is due to the following pair connections, respectively: #4−Glu−#23, #5−Asp−#21, #9−Cys−#18, #19−His−#22, #20−Gln−#28, #23−Phe−#32, #24−Tyr−#29, #26−Asn−#30, #27−Lys−#32. The degeneracies 3 for Ile and 1 for Met are due to the route duality #21−Ile−#30∼#22−Met/Ile−#29. The degeneracy 1 for Trp satisfies the pair connection with non-standard genetic code #12−Trp/stop(Trp)−#15. This pair connection includes a stop codon; the other stop codons satisfy the pair connection: #25−stop−#31. Incidentally, the route dualities for non-standard codons are also due to recognition of non-standard tRNAs by the corresponding aaRSs: #8−Ser−#17∼#11−(Ser)−#14, #4−(Thr)−#27∼#20−(Thr)−#25, #11−(stop)−#14∼#15−stop−#31.

## 4. Conclusions

In the present prebiotic picture with selective pressure, both the codon degeneracy and the major/minor groove classification of aaRSs have been explained together within the scope of literature.

## Figures and Tables

**Table 1:** Selective pressure due to the unique roadmap with increasing stability.

Stability	CG*N	GC*N	TA*N	AT*N
(−)		GC*A		AT*C AT*A
(+)	CG*G	GC*C GC*G	TA*C TA*G TA*A	AT*T
(++)	CG*A CG*T	GC*T		
(3+)				AT*G
(4+)	CG*C		TA*T	
	(+)CG*G → (++)CG*A increase in stability	(+)GC*C → (−)GC*A *unstable*	(+)TA*A → (+)TA*G *no increase in stability*	(+)AT*T → (3+)AT*G
	(+)CG*G → (4+)CG*C increase in stability	(+)GC*C → (+)GC*G *no increase in stability*	(+)TA*A → (4+)TA*T	(+)AT*T → (−)AT*A *unstable*
	(+)GC*C → (++)GC*T increase in stability	(+)CG*G → (++)CG*T	(+)AT*T → (+)AT*C *no increase in stability*	(+)TA*A → (+)TA*C *no increase in stability*
	POSSIBLE (Roadmap)	Impossible	Impossible	Impossible
	(+)CG*G → (++)CG*T	(+)GC*C → (++)GC*T	(+)TA*A → (+)TA*C *no increase in stability*	(+)AT*T → (−)AT*C *unstable*
	(+)CG*G → (4+)CG*C	(+)GC*C → (+)GC*G *no increase in stability*	(+)TA*A → (4+)TA*T	(+)AT*T → (−)AT*A *unstable*
	(+)GC*C → (−)GC*A *unstable*	(+)CG*G → (++)CG*A	(+)AT*T → (3+)AT*G	(+)TA*A → (+)TA*G *no increase in stability*
	Impossible	Impossible	Impossible	Impossible

**Table 2:** Formation of the codon boxes via (quasi) route dualities.

	Hierarchy 1 to Hierarchy 2				Hierarchy 2 to Hierarchy 3								Hierarchy 3 to Hierarchy 4			
Route 0	1Gly		6Pro		3Glu		7Ser	8Leu		10Arg				17Phe		20Lys
Route 1	1Gly	2Ala			4Asp	5Val			9Thr	7Ser			15Ile			19Asn
Route 2		2Ala		10Arg		5Val			9Thr		11Cys	13His	16Met		18Tyr	
Route 3			6Pro	10Arg			7Ser	8Leu			12Trp	14Gln		8Leu	stop	
Codon box	GGN	GCN	CCN	CGN	GAN	GUN	UCN	CUN	ACN	AGN	UGN	CAN	AUN	UUN	UAN	AAN

## Supplementary Materials

The following are available online at https://www.mdpi.com/article/10.3390/genes12122023/s1, Movie S1: Substitutions of triplex base pairs along the roadmap.
